# Direct and bisulfite-free 5-methylcytosine and 5-hydroxymethylcytosine sequencing at single-cell resolution with scTAPS and scCAPS + 

**DOI:** 10.1186/s13059-025-03708-1

**Published:** 2025-08-18

**Authors:** Xiufei Chen, Jingfei Cheng, Linzhen Kong, Xiao Shu, Haiqi Xu, Masato Inoue, Marion Silvana Fernández-Berrocal, Dagny Sanden Døskeland, Magnar Bjørås, Shivan Sivakumar, Yibin Liu, Jing Ye, Chun-Xiao Song

**Affiliations:** 1 Department of Central Laboratory, The First Affiliated Hospital of Ningbo University, Ningbo, 315010 China; 2 Nuffield Department of Medicine, Ludwig Institute for Cancer Research, University of Oxford, Oxford, OX3 7FZ UK; 3 Target Discovery Institute, Nuffield, Department of Medicine , University of Oxford, Oxford, OX3 7FZ UK; 4 Department of Clinical and Molecular Medicine (IKOM), Norwegian University of Science and Technology (NTNU), 7491 Trondheim, Norway; 5 Department of Microbiology, Oslo University Hospital, University of Oslo, 0424 Oslo, Norway; 6 Centre for Embryology and Healthy Development, University of Oslo, 0373 Oslo, Norway; 7 The Institute of Immunology and Immunotherapy, College of Medical and Dental Sciences, University of Birmingham, Edgbaston, Birmingham, B15 2TT UK; 8 Kennedy Institute of Rheumatology, University of Oxford, Oxford, OX3 7FY UK; 9 State Key Laboratory of Metabolism and Regulation in Complex Organisms, College of Chemistry and Molecular Sciences, Taikang Center for Life and Medical Sciences, Wuhan University, Wuhan, 430072 China

**Keywords:** Single-cell sequencing, 5mC, 5hmC, scTAPS, scCAPS +, Aging, Neuron

## Abstract

We present direct sequencing methodologies, scTAPS for 5-methylcytosine (5mC) and 5-hydroxymethylcytosine (5hmC) and scCAPS + specifically for 5hmC, enabling quantitative detection of 5mC and 5hmC at single-base resolution and single-cell level. Achieving approximately 90% mapping efficiency, our plate-based methods accurately recover 5mC and 5hmC profiles in CD8 + T and mouse embryonic stem cells. Notably, scCAPS + reveals a global increase in 5hmC across neuronal and non-neuronal cells in the hippocampus of aging mice. Our methods offer strong potential for seamless integration into high-throughput single-cell multi-omics, facilitating future investigations of epigenomic dynamics in specific biological processes.

**Supplementary Information:**

The online version contains supplementary material available at 10.1186/s13059-025-03708-1.

## Background

5-Methylcytosine (5mC) is a prevalent epigenetic modification in mammalian DNA, which is present in approximately 70–80% of the symmetrical CpG dinucleotides [1]. A major derivative of 5mC is 5-hydroxymethylcytosine (5hmC), formed by TET oxidation [2]. Over the last two decades, extensive research has focused on elucidating distinct roles of 5mC and 5hmC in various biological processes [3, 4]. To map 5mC and 5hmC at single-cell resolution, several sequencing methods based on bisulfite sequencing (BS-Seq) have been developed [5–9]. However, these methods encounter challenges such as substantial DNA damage and limited mapping efficiency. Recently, bisulfite-free methods [10–12] have emerged as alternatives to single-cell BS-Seq (scBS-Seq). It is worth noting that these methodologies still employ an indirect approach by converting unmodified cytosine (uC), resulting in low sequence complexity. Notably, several newly developed methods, such as six-letter sequencing [13] and SIMPLE-seq [14], have recently emerged, allowing the simultaneous detection of both 5mC and 5hmC. Previously, we developed TET-assisted pyridine borane sequencing (TAPS) [15] and chemical-assisted pyridine borane sequencing plus (CAPS +) [16, 17] for direct 5mC and 5hmC detection, respectively, but at the bulk level.

## Results

**Fig. 1:**
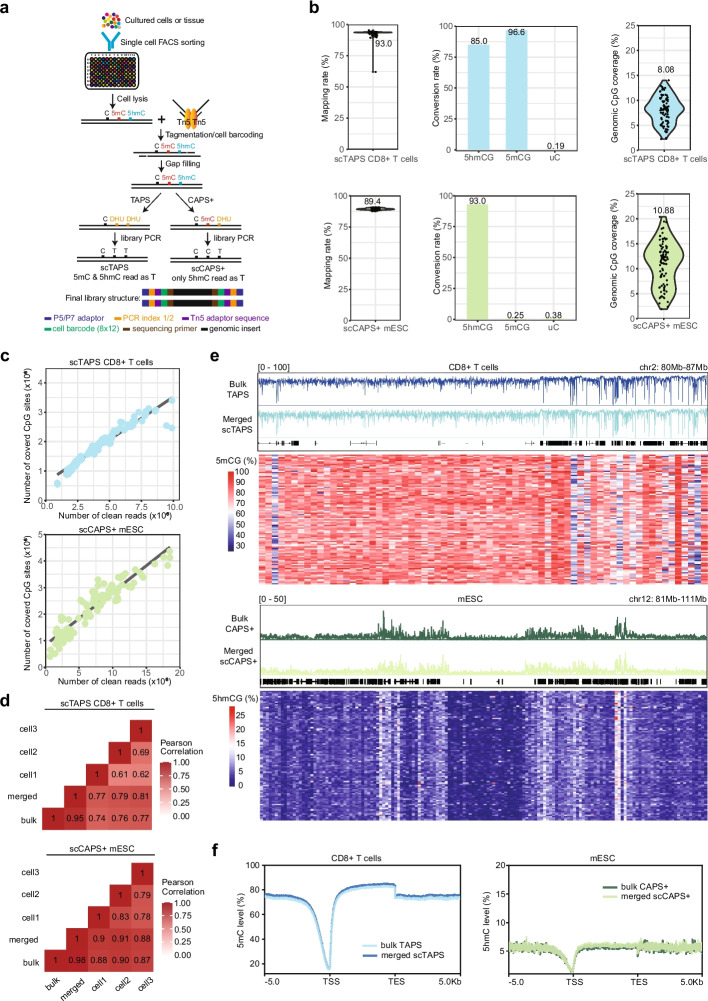
Validation of scTAPS and scCAPS +. **a** Overview of the scTAPS and scCAPS + methodologies. **b** Violin plots showing the mapping rates (left) and genomic CpG coverage (right). Each point represents a single cell (96 mESC and 96 CD8 + T cells in total). Barplots (middle) show conversion rates based on 5hmCG, 5mCG, and false positives on unmodified C spike-ins. The values above represent the means. Top: scTAPS (CD8 + T cells). Bottom: scCAPS + (mESC). **c** Saturation curve showing the number of covered CpG sites and the number of cleaned reads sequenced in each cell. **d** Heatmap showing Pearson correlation coefficient between bulk and single cell TAPS (CD8 + T cells)/CAPS + (mESC). **e** 5mCG and 5hmCG level along chr2:80,000,000–87,000,000 in CD8 + T cell (top) and 5hmCG level along chr12:81,000,000–111,000,000 in mESC (bottom). IGV tracks in upper panel showing bulk 5mCG/5hmCG and merged 5mCG/5hmCG signals from scTAPS/scCAPS +. Heatmap in lower panels showing 5mCG/5hmCG levels in 100 kb/200 kb bins (column) across 96 CD8 + T cells/mESC (row) in scTAPS/scCAPS +. Level is scaled by color. **f**. Metagene plots showing 5mCG (CD8 + T cells) and 5hmCG (mESC) distribution along gene body from 5 kb upstream of TSS (Transcription Start Sites) to 5 kb downstream of TES (Transcription End Sites)

In this study, we introduce single-cell TAPS (scTAPS) and single-cell CAPS + (scCAPS +) by integrating Tn5 transposon-based fragmentation with TAPS and CAPS +, enabling robust and precise profiling of DNA 5mC and 5hmC, respectively, at the single-cell level in a bisulfite-free manner. Following an assessment of the feasibility of low input TAPS and CAPS + (Additional file 1: Fig. S1), we adopted our methodology for single-cell analysis after further optimization, including investigating the impact of Tn5 tagmentation temperature on library size (Additional file 1: Fig. S2). We first validated our single-cell methods in human peripheral blood CD8 + T cells for scTAPS and mouse embryonic stem cells (mESC) for scCAPS + (Fig. 1, Additional file 2: Table S1, 2). Briefly, individual cells or nuclei are sorted via fluorescence-activated cell sorting (FACS), and then undergo lysis and fragmentation using barcoded Tn5 transposons. After gap-filling and DNA purification, barcoded DNA from 96 cells is pooled, and subsequently TAPS or CAPS + reactions are employed (Fig. 1a). Compared to previous methods [5–7, 10, 11], we achieved notably high mapping efficiency in scTAPS (93.0%) and scCAPS + (89.4%) (Fig. 1b), surpassing those of other indirect methodologies (Additional file 1: Fig. S3a). Notably, using incorporated spike-in controls, we observed robust conversion rates in both scTAPS (5mCG: 96.6%, 5hmCG: 85.0%) and scCAPS + (5hmCG: 93.0%), along with very low false positive rates in scTAPS (uC: 0.19%) and scCAPS + (uC: 0.38%, 5mCG: 0.25%) (Fig. 1b). With a sequencing depth as low as 4.8 and 7.7 million 120-base-pair (bp) paired-end reads per single cell, we achieved a mean coverage of 2.0 and 2.3 million CpG sites (8.08% and 10.88% of the total CpG sites) in scTAPS and scCAPS +, respectively (Fig. 1b). Our approach exhibits greater CpG and genomic coverage than other published methods at comparable sequencing depths (Additional file 1: Fig. S3b). We note that increased sequencing depth is likely to improve genomic coverage (Fig. 1c). Furthermore, 5mC/5hmC profiles in bulk TAPS and CAPS + correlated strongly with 96 merged single cells (Pearson’s *r* = 0.95 and 0.98) and 3 individual cell representatives (Fig. 1d). The average correlation of methylation between individual single cells and bulk data is approximately 0.700 for scTAPS and 0.785 for scCAPS +, which can be further improved by increased sequencing depth (Additional file 1: Fig. S4a, b). The single-cell 5mC/5hmC level closely aligned with those in the bulk samples (Fig. 1e). Additionally, a concurrent reduction in both 5mC and 5hmC was observed in the proximity of transcription start site (TSS) regions (Fig. 1f, Additional file 1: Fig. S5a, b), consistent with low input results and previous findings [15, 17]. Taken together, these findings highlighted the accuracy of scTAPS and scCAPS + in mapping 5mC and 5hmC at the single-cell level.

**Fig. 2:**
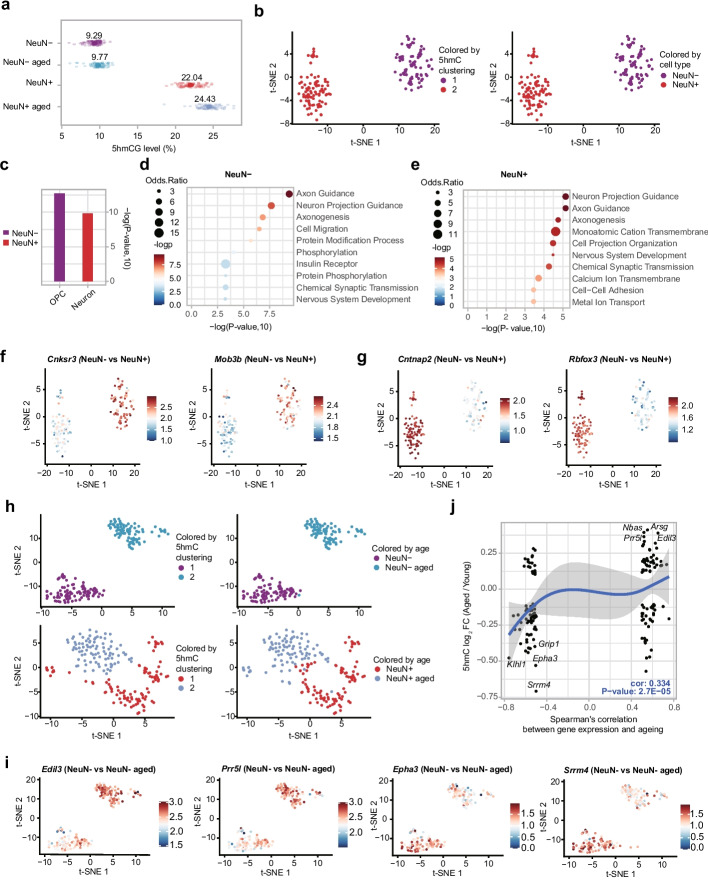
scCAPS + reveals the 5hmC dynamics in hippocampus neuron and non-neuron cells during aging. **a** Global 5hmC levels in young and aged non-neurons (NeuN-) or neurons (NeuN +). **b** t-SNE plot showing the 5hmC clustering of young non-neurons and neurons. **c** The top enriched cell ontology determined based on marker genes using the Tabula Muris dataset. OPC: Oligodendrocyte Progenitor Cell. **d**,** e** Top 10 enriched GO terms for both non-neuron (**d**) and neuron (**e**) marker genes. **f**,** g** t-SNE plot showing 5hmC levels of non-neurons marker genes (**f**) *Cnksr3* and *Mob3b* and neurons marker genes (**g**) *Cntnap2* and *Rbfox3*. The scale bar represents the normalized gene body hmCG level. **h** t-SNE plot showing age-related 5hmC clustering in both non-neuron (top) and neuron (bottom) cells. **i** t-SNE plot showing 5hmC levels of *Edil3* and *Prr5l*,* Epha3*, and *Srrm4*, in young and aged non-neurons. **j** The scatter plot showing the 5hmC changes in non-neurons during aging, along with the corresponding Spearman’s correlation coefficient depicting the relationship between gene expression and aging in the mouse brain. Pearson correlation coefficient and significance level were computed

The hippocampus plays a pivotal role in learning and memory [18, 19], and 5hmC has been found specifically enriched in the brain region[20]. To unravel aging-related 5hmC dynamics in this region at the single-cell level, neurons and non-neurons were isolated from young (3 months) or aged (18 months) mice, followed by scCAPS + analysis (Additional file 1: Fig. S6a, b, Fig. S7a, b, Additional file 3: Table S1-5). We found hippocampus neurons exhibited higher 5hmC levels compared to non-neurons (22.04% vs 9.29%) (Fig. 2a). Utilizing gene body 5hmC levels, two distinct clusters (cluster 1 and cluster 2) were identified, closely corresponding to FACS-identified non-neuron and neuron cells (Fig. 2b). Next, we employed marker genes defined by 5hmC levels to annotate the two clusters using the Tabula Muris database [21]. This analysis revealed that cluster 1 corresponds to Oligodendrocyte Precursor Cells (OPC) (CL: 0002453), while cluster 2 corresponds to neuronal cells (CL: 0000540) (Fig. 2c). Gene Ontology (GO) analysis showed the enrichment of functions related to axon guidance and neuron projection guidance in both neuron and non-neuron marker genes (Fig. 2d, e), suggesting the significance of both cell types in maintaining fundamental neuronal functions. Non-neuron marker genes (*Cnksr3*,* Mob3b*,* Sema4d*,* Dock5*)[22] exhibited significantly higher 5hmC levels in non-neurons, while neuron marker genes (*Cntnap2*,* Rbfox3*,* Syt1*,* Grm1*) [22] displayed markedly elevated 5hmC levels in neurons (Fig. 2f, g, Additional file 1: Fig. S7c, and Additional file 3: Table S2). Moreover, distinct separations between young and aged cells were observed in both non-neurons and neurons based on the 5hmC signal (Fig. 2h, Additional file 3: Table S3, 4). Correlation analysis revealed that genes exhibiting a positive correlation between expression and age [23] (e.g., *Edil3* and *Prr5l*,* Galntl6* and *Atg10*) tended to have higher levels of 5hmC in aged cells, and vice versa (e.g., *Epha3 *and* Srrm4*,* Eps8* and *Acvr1*), particularly in non-neurons (Fig. 2i–j, Additional file 1: Fig. S7d, e). However, certain genes, such as *App*, exhibit opposite patterns of 5hmC change, with an increase observed in aged non-neuronal cells but a decrease in aged neurons (Additional file 1: Fig. S7f).

## Discussion

Compared to other indirect methods, our methods do not need specially modified (C-depleted or 5mC/5hmC modified) Tn5 adaptor sequences [8, 10]. Direct conversion of 5mC and 5hmC to T will preserve the genomic complexity, thus leading to high sequencing quality. During manuscript preparation, the SIMPLE-seq method was published, incorporating our TAPS approach for single-cell DNA methylation detection [14]. However, compared to our method, SIMPLE-seq exhibited lower conversion rates (~ 87% for 5mC), necessitating the use of a standard curve for modification level adjustment. Additionally, it exhibited significantly lower genome coverage (1.96% for 5mC and 0.79% for 5hmC) likely attributed to its high-throughput capacity which compromises sequencing depth per cell. Our plate-based approach achieves high coverage at comparable sequencing depths. This strategy could complement high-throughput, low-coverage approaches to serve distinct biological contexts and applications, much like the complementary roles of Smart-seq3 [24] and 10X Genomics platforms [25]. Future adaptation of scTAPS and scCAPS + for high-throughput formats could help overcome current limitations in cell numbers, providing more detailed insights at single-cell resolution. The strengths and limitations of our methods become evident when compared to various current methods [7, 10, 13, 14] capable of detecting both 5mC and 5hmC modifications (Additional file 4: Table S1).

## Conclusions

In summary, we introduce scTAPS and scCAPS + as robust techniques for the precise and direct detection of 5mC and 5hmC in single cells. By applying scCAPS + to mouse hippocampus, we revealed dynamics of 5hmC during aging.

Considering the robust efficiency and accuracy of scTAPS/scCAPS +, we anticipate that our methodologies will facilitate future investigations into the roles of 5mC and 5hmC in specific biological processes. Future development of our methods holds promise for seamless integration into high-throughput single-cell multi-omics sequencing for simultaneous genomic and epigenomic analysis. Moreover, they offer potential applicability in spatial DNA modification sequencing [26].

## Methods

### Human CD8 + T cells

Human CD8 + T cells were isolated from blood cones by negative immunodensity selection using CD8 + (RosetteSep, StemCell Technologies, #15,023). Cells were then single-cell sorted on a BD Aria III into 96-well plates.

### mES cell culture

E14 mouse embryonic stem cells (mESC, a gift from S. Kriaucionis, University of Oxford) were cultured on gelatin-coated plates in DMEM (Invitrogen 11,995) supplemented with 15% FBS (Gibco 16,010,159), 2 mM L-glutamine (Gibco 25,030), 1% non-essential amino acids (Gibco 11,140), 1% penicillin/streptavidin (Gibco 15,140), 0.1 mM β-mercaptoethanol (Gibco 21,985), 1000 units/ml LIF (leukaemia inhibitory factor) (Millipore ESG1107), 1 μM PD0325901 (Stemgent 04–0006), and 3 μM CHIR99021 (Stemgent 04–0004). The cells were maintained at 37 °C and 5% CO_2_. Although the cell lines were not authenticated, they tested negative for mycoplasma contamination. Before cell sorting, the cells were harvested by centrifugation and passed through a 40 μm cell strainer (Falcon) to achieve single-cell suspension.

### Animals

3 and 18 months old male mice with the C57BL/6N background were used in the study. Animals were housed with their littermates in 1717 × 545 × 2045 mm (LxWxH) cages with free food and water access in a dedicated room (temperature 22 °C ± 1 °C and humidity 55% ± 5%) with a 12 h light/dark cycle (lights on 7 pm to 7 am). All animal experiments were conducted in accordance with the Norwegian Animal Welfare Act and approved by the Norwegian Animal Research Authority (FOTS 28340).

### Hippocampal tissue collection and nuclei isolation

Mice were anesthetized using isoflurane (Baxter, Oslo, Norway) and subsequently killed by an intraperitoneal overdose of pentobarbital (> 200 mg/kg body weight). The mouse brain was directly extracted without intracardial perfusion, and the hippocampal regions were micro-dissected in cold PBS. Nuclei from freshly dissected hippocampus (HPC) were isolated with EZ PREP kit (Sigma, Cat #NUC-101). Briefly, two single-side HPC from two mice at 3 m or 18 m age were resuspended in 1 ml ice-cold EZ lysis buffer, homogenized using a glass Dounce tissue grinder (15 times with the loose pestle and 15 times with the tight pestle), and incubated on ice for 5 min. Homogenate was strained through a 30-μm cell strainer (Miltenyi Biotech) and centrifuged at 850 × g for 10 min (4 °C) to pellet nuclei. Nuclei were washed in 1 ml ice-cold EZ lysis buffer with a 5-min incubation on ice, and then pelleted by centrifugation (850 × g, 10 min, 4 °C).

### Nuclei staining and Flow cytometry-based cell sorting (FACS)

Nuclei were resuspended in 1 ml staining buffer (1 × PBS supplemented with 1% nuclease-free BSA) and incubated at 4 °C for 10 min (blocking). Mouse anti-NeuN antibody (Merck Millipore, MAB377, Clone A60, RRID: AB_2298772) was added to the nuclei at a final dilution of 1:500, and nuclei suspensions were incubated at 4 °C for 30 min. Nuclei were pelleted with centrifugation (450 × g, 10 min, 4 °C) and washed once in the staining buffer. After washing and pelleting, nuclei were resuspended in 1 ml staining buffer containing the secondary antibody (goat anti-mouse IgG1, Alexa Fluor™ 488, ThermoFisher Scientific, RRID: AB_2535764) at a final dilution of 1:1000. After a 30-min incubation at 4 °C, nuclei were again pelleted (450 × g, 10 min, 4 °C) and washed once in the staining buffer. Prior to FACS, nuclei were resuspended in 1 ml staining buffer containing DAPI at a final concentration of 0.1ug/ml and filtered through a 30-μm cell strainer. Single nuclei were captured by gating on DAPI-positive events, and then gating on Alexa Fluor 488 (NeuN) signal. NeuN + and NeuN- nuclei were sorted in Optical 96-Well plates (ThermoFisher Scientific) containing 4 µl of lysis buffer consisting of 16.67 mM TAPS-NaOH buffer (pH 8.5, Alfa Aesar, Cat: J63268), 8.33 mM MgCl_2_(Invitrogen), 0.67 × NEB buffer 4 (NEB, B7004S), 0.13% Triton X-100 (sigma, X100-500ML), and 1 μg Qiagen protease in each well.

### The preparation of Spike-ins and 2 kb filler DNA for scTAPS and scCAPS + 

Fully methylated lambda phage DNA, 2 kb unmodified DNA, and 2 kb filler DNA were made as previously described [15, 17]. To make 5hmC spike-in, three DNA oligos (5 μM each) (Additional file 5: Table S1) from IDT were annealed in 1 × New England Biolabs (NEB) buffer 2 (20 μl in total). Subsequently, the annealed oligos were incubated with 10 mM 5-hydroxymethyl-2′-dCTP (Zymo Research), along with dGTP, dATP, dTTP (NEB), and 10 U Klenow exo^–^ (M0212L) in a 50-μl reaction at 37 °C for 1 h. T4 ligation was then carried out at room temperature for 30 min. Following purification, the DNA underwent End Repair and A-Tailing (ER&T), followed by ligation with Tn5 i7/i5 adaptors sequence with T in the 3′ end. The resulting spike-in sequence (Additional file 5: Table S2) was finally purified using 1.8 × AMPure XP beads, following the provided guidelines.

### Tn5 protein purification

Tn5 protein is purified as previously described with some modifications [27], NEB C3013 cells were transformed with the pTBX1-Tn5 plasmid. Tn5 protein is induced by adding 250 µl of 1 M IPTG ((Isopropyl β-D-1-thiogalactopyranoside) into 1 L LB culture when the A600 reached 0.7–0.9, followed by incubation for 16–18 h at 16 °C in a shaking incubator. The cells were pelleted at 4000 rpm for 30 min and resuspended in 50 mL of cold HEGX buffer (20 mM HEPES–KOH pH 7.2, 0.8 M NaCl, 1 mM EDTA, 10% glycerol, 0.2% Triton X-100, 1 × complete Protease Inhibitor Cocktail). Cell lysate was sonicated with a BioRuptor sonicator for 20 min at 40% Amp, 5 s on/10 s off (on ice) and then centrifuged at 35,000* g* for 60 min at 4 °C. The supernatant was collected and precipitated with 2.1 mL of 10% neutralized PEI in a dropwise manner. The resulting mixture was centrifuged at 21,500* g* for 10 min at 4 °C, and the supernatant was pre-incubated with 10 mL of washed chitin beads for 3 h in a cold room. The sample was loaded onto a gravity column, washed with 500 mL of cold HEGX buffer, and equilibrated with 5 mL of HEGX with 100 mM DTT (Dithiothreitol). The column was incubated for Tn5 elution at 4 °C for 48 h. The eluted sample was dialyzed overnight at 4 °C in 2 L of 2 × Tn5 dialysis buffer (100 mM HEPES–KOH pH 7.2, 0.2 M NaCl, 0.1 mM EDTA, 20% glycerol, 2 mM fresh added DTT) using a Spectra/Por 6-8kD dialysis membrane. The dialyzed sample was quantified for concentration using Nanodrop, mixed with an equal volume of 100% glycerol for homogeneity, frozen in liquid nitrogen, and stored in aliquots at − 80 °C before use.

### Tn5 transposome assembly

96 combinatory Tn5 assembly were performed as described before [27], and their sequences are presented herein. Briefly, 8 × 12 Tn5MEDS-i5/i7 and Tn5MEDS-Rev oligos [28] were purchased from IDT (Additional file 5: Table S3), and annealing was conducted in 1 × TE buffer with a 25 μl total volume (95 °C for 5 min, followed by a gradual decrease in temperature at a rate of 1 °C per 12 s for 180 cycles, and cooling down to 4 °C). The Tn5 assembly took place in a 96-well plate, combining 2 μL of Tn5 (10 ng/μL), 2 μL of pre-annealed Tn5MEDS-i5 and i7 oligos, 25 μL of glycerol, and 29 μL of 2 × Tn5 dialysis buffer to reach a total volume of 60 μL. This reaction was incubated at room temperature for 1 h, followed by a sevenfold dilution to produce 1 × preassembled Tn5 complexes. Preassembled Tn5 complexes were stored at − 80 °C and demonstrated stability over a one-month period. 1 μL of 1 × preassembled Tn5 was always added to each well for scTAPS and scCAPS + experiment.

### mTET1 protein expression

The mTET1 purification is conducted as previously described [15]. Briefly, the mTet1CD catalytic domain (NM_001253857.2, 4371–6392) was integrated into the pcDNA3-Flag vector using KpnI and BamH1 restriction sites. Subsequently, 1 mg of plasmid was transfected into 1L of Expi293F cell culture at a density of 1 × 10^6^ cells ml^−1^. The cells were cultured for 48 h at 37 °C, 170 r.p.m., and 5% CO2. Following incubation, cells were harvested by centrifugation, resuspended in a lysis buffer (50 mM Tris–Cl pH 7.5, 500 mM NaCl, 1 × complete Protease Inhibitor Cocktail, 1 mM PMSF, 1% Triton X-100), and incubated on ice for 20 min. Next, the lysed cell solution was clarified by centrifugation at 30,000* g* and 4 °C for 30 min. The resulting supernatant was purified using ANTI-FLAG M2 Affinity Gel. Pure protein was eluted in the buffer (20 mM HEPES pH 8.0, 150 mM NaCl, 0.1 mg ml^−1^ 3 × Flag peptide, 1 × complete Protease Inhibitor Cocktail, and 1 mM PMSF). The collected fractions were then concentrated, andbuffer exchanged into a final buffer of 20 mM HEPES pH 8.0, 150 mM NaCl, and 1 mM DTT. The concentrated protein was mixed with glycerol (30% v/v), frozen in liquid nitrogen, and stored in aliquots at − 80 °C.

### scTAPS and scCAPS + 

For scTAPS, 96 cells were pooled for DNA purification and subjected to mTET1 oxidation as previously described [15]. A reaction mixture of 50 μl was prepared, comprising 50 mM HEPES buffer (pH 8.0), 100 μM ammonium iron (II) sulfate, 1 mM α-ketoglutarate, 2 mM L-ascorbic acid, 2.5 mM DTT, 1.2 mM ATP, 100 mM NaCl, and 4 μM mTet1CD. The reaction was conducted at 37 °C for 80 min. Subsequently, 2.0 U of Proteinase K (New England Biolabs) was introduced to the reaction mixture and incubated at 50 °C for 1 h to stop the oxidation process. The oxidized DNA was purified using 1.8 × AMPure XP beads, following the provided guidelines. To ensure complete oxidation, a second round of oxidation was performed. The double-oxidized DNA was purified and then underwent borane reduction and purification using a Zymo column with Oligo Binding Buffer.

For scCAPS +, 96 cells were pooled for DNA purification and subjected to a series of chemical reactions to convert 5hmC into 5fC and then to 5caC as previously described with some modifications [17]. DNA were incubated with 4-acetamido-2,2,6,6-tetramethylpiperidine-1-oxoammonium tetra-fluoroborate (ACT + BF_4_-) in a 25 μl solution comprising sodium phosphate buffer at 25 °C for 16 h. The resultant oxidized DNA was purified using 1.8 × AMPure XP Beads. Then Pinnick oxidation was conducted in a 30 μl reaction mixture containing sodium acetate buffer, NaClO_2_, and 2-methyl-2-butene at 25 °C for 16 h. The purified product then underwent borane reduction and purification using a Zymo column with Oligo Binding Buffer.

### scTAPS and scCAP + library construction and sequencing

Individual cells were isolated into a 96-well plate through FACS as mentioned above. Following centrifugation at 2000 rpm for 5 min, the plates were either preserved at − 80 °C or progressed to subsequent procedures. Single cells were lysed by incubating at 50 °C for 3 h, followed by incubation at 75 °C for 30 min, 80 °C for 15 min, and a 4 °C hold. Tagmentation was performed at 50 °C for 15 min in a reaction mix consisting of 1 × TAPS buffer (10 mM TAPS-NaOH, 5 mM MgCl_2_), 6% PEG (polyethylene glycol) 3,350, 1 µL 1 × preassembled Tn5, and H_2_O to a total volume of 10 μ. Tn5 was stripped off by adding 1.11 μL of 1.0% SDS (Sodium Dodecyl Sulfate), followed by incubating at room temperature for 5 min. Neutralization was performed by adding 1.24 μL of 5.0% NP40 or Triton X-100, followed by incubating at room temperature for 5 min. Subsequently, 12.35 μL of 2 × Phusion High-Fidelity PCR Master Mix with HF Buffer (Thermo Scientific, F531L) was added, followed by incubating at 72 °C for 5 min, and a 4 °C hold. The 96 reactions were combined into a 50-mL BD tube and supplemented with 2-kb filler DNA (200 ng) and 0.1% spike in DNAs. Following purification, the TAPS or CAPS + reaction was employed as mentioned, and the final sequencing library was amplified utilizing the KAPA HiFi HotStart Uracil + ReadyMix PCR Kit along with index primers from the Nextera XT Index Kit (Illumina, FC-131–1001). The amplification protocol involved an initial step at 98 °C for 45 s, followed by 13 cycles (98 °C for 10 s, 60 °C for 15 s, and 72 °C for 1 min), then incubation at 72 °C for 3 min, 4 °C hold. The library was subjected to purification and size selected to 500–700 bp by 0.5 × –0.25 × Ampure XP beads. Custom sequencing primers are used for custom Nova Sequencing (Additional file 5: Table S4).

### Data analysis for scTAPS and scCAPS + 

Pre-processing: Raw sequenced reads were demultiplexed using the demuxbyname2.sh script from BBMap (version 38.50; https://www.osti.gov/biblio/1241166). Demultiplexed reads were processed with fastp [29] package using default parameters to remove low-quality bases. The trimmed reads were aligned to the reference genome using bwa [30] with default parameters. Reference sequence for the mouse genome was downloaded from https://hgdownload.cse.ucsc.edu/goldenpath/mm9/bigZips/; reference sequence for human CD8 + T-cell was downloaded from: ftp://ftp.ncbi.nlm.nih.gov/genomes/all/GCA/000/001/405/GCA_000001405.15_GRCh38/seqs_for_alignment_pipelines.ucsc_ids/GCA_000001405.15_GRCh38_no_alt_analysis_set.fna.gz . Uniquely mapped reads were filtered using samtools [31] tools with MAPS ≥ 10. PCR duplicates were called using Picard (2.23.0-Java-11) Mark Duplicates (https://broadinstitute.github.io/picard/). Methylation was called using a custom R script used in our previous project [32]. 10 bp in start or end of reads were excluded for methylation calling. Snakemake [33] pipeline file with detailed steps is provided in the code availability section.

Clustering for mouse hippocampus scCAPS + : Cells with an insufficient number (< 500,000) or an excessively high count (> 3,000,000) of properly mapped reads were systematically excluded from downstream analysis, while properly mapped reads were defined as reads mapped after using MAPQ > 1 and Picard’s MarkDuplicates filtering step. The 5hmCG level for each annotated protein-coding gene was computed by dividing the sum of methylated base calls by the total base calls across the gene body. Gene annotation was downloaded from https://ftp.ebi.ac.uk/pub/databases/gencode/Gencode_human/release_43/gencode.v43.basic.annotation.gtf.gz. Genes with total calls less than 5 were excluded for analysis. The Seurat [34] package was used for downstream analysis with parameters specified in the code availability section for reproducibility-related details. Specifically, using gene body 5hmCG level as input, we performed an unbiased clustering using the FindClusters function in Seurat. To determine marker genes, we employed the FindMarkers function in Seurat using the min.pct = 0.25 parameter. This analysis identified genes with differential 5hmCG levels between the populations. For subsequent functional enrichment analysis, genes with an adjusted *p*-value (p_val_adj) less than 0.05 were selected. The enrichment analysis was conducted using the enrichR package [35] against the GO_biological_Process_2023 ontology and the Tabula_Muris database (https://tabula-muris.ds.czbiohub.org/). The age-correlated genes and the correlation between their expression and age were downloaded from a previous study [23].

Annotation: Annotated genes were downloaded from the RefSeq database (http://hgdownload.soe.ucsc.edu/goldenPath/mm9/bigZips/genes/mm9.refGene.gtf.gz) for mESCs and the Gencode database (https://ftp.ebi.ac.uk/pub/databases/gencode/Gencode_human/release_43/gencode.v43.basic.annotation.gtf.gz) for CD8 + T cells. 5mCG/5hmCG scores within gene bodies, 5 kb upstream of TSS, and 5 kb downstream regions of transcription termination sites (TTS) were calculated by computeMatrix in deepTools (v.3.3.1) [36] with parameters (–beforeStartLength 5000 –regionBodyLength 5000 –afterRegionStartLength 5000 –binSize 10). The plots were created with the plotProfile function. Chromatin states for mESC and CD8 + cells were downloaded from https://github.com/gireeshkbogu/chromatin_states_chromHMM_mm9 [37] and https://github.com/ernstlab/full_stack_ChromHMM_annotations [38], respectively. Aggregated 5mC/5hmC signals were calculated in each chromatin state in each cell.

## Supplementary Information

Additional file 1: Fig. S1-S7. Supplementary figures

Additional file 2: Quality Control for scTAPS in CD8 + T Cells and scCAPS + in mESC

Additional file 3: scCAPS + analysis in young and aged neurons and non-neurons. 

Additional file 4: Comparison of the advantages and disadvantages of scTAPS/CAPS + with other methods for detecting both 5mC and 5hmC. 

Additional file 5: Oligo sequences

Additional file 6: Review history

## Data Availability

Raw and processed data associated with this study were deposited in the Gene Expression Omnibus (GEO) database under accession: GSE255771[39]. The source code (MIT license) for bioinformatics analysis of the paper is available on GitHub repository (https://github.com/jfeicheng92/scTAPS_CAPS) [40] and Zenodo (10.5281/zenodo.14039493)[41].
