# Supplementary material for: Direct and bisulfite-free 5-methylcytosine and 5-hydroxymethylcytosine sequencing at single-cell resolution with scTAPS and scCAPS +
Source: Genome Biol. 2025 Aug 18;26:244. doi: 10.1186/s13059-025-03708-1 (PMC12359873; doi:10.1186/s13059-025-03708-1)
Supplement: Supplementary file 1 — Additional file 1: Fig. S1-S7. Supplementary figures [file 13059_2025_3708_MOESM1_ESM.pdf]

Direct and bisulfite-free 5-methylcytosine and 5-hydroxymethylcytosine sequencing at single-cell resolution with scTAPS and scCAPS+

Additional file 1: Fig. S1-S7. Supplementary figures.

Fig. S1

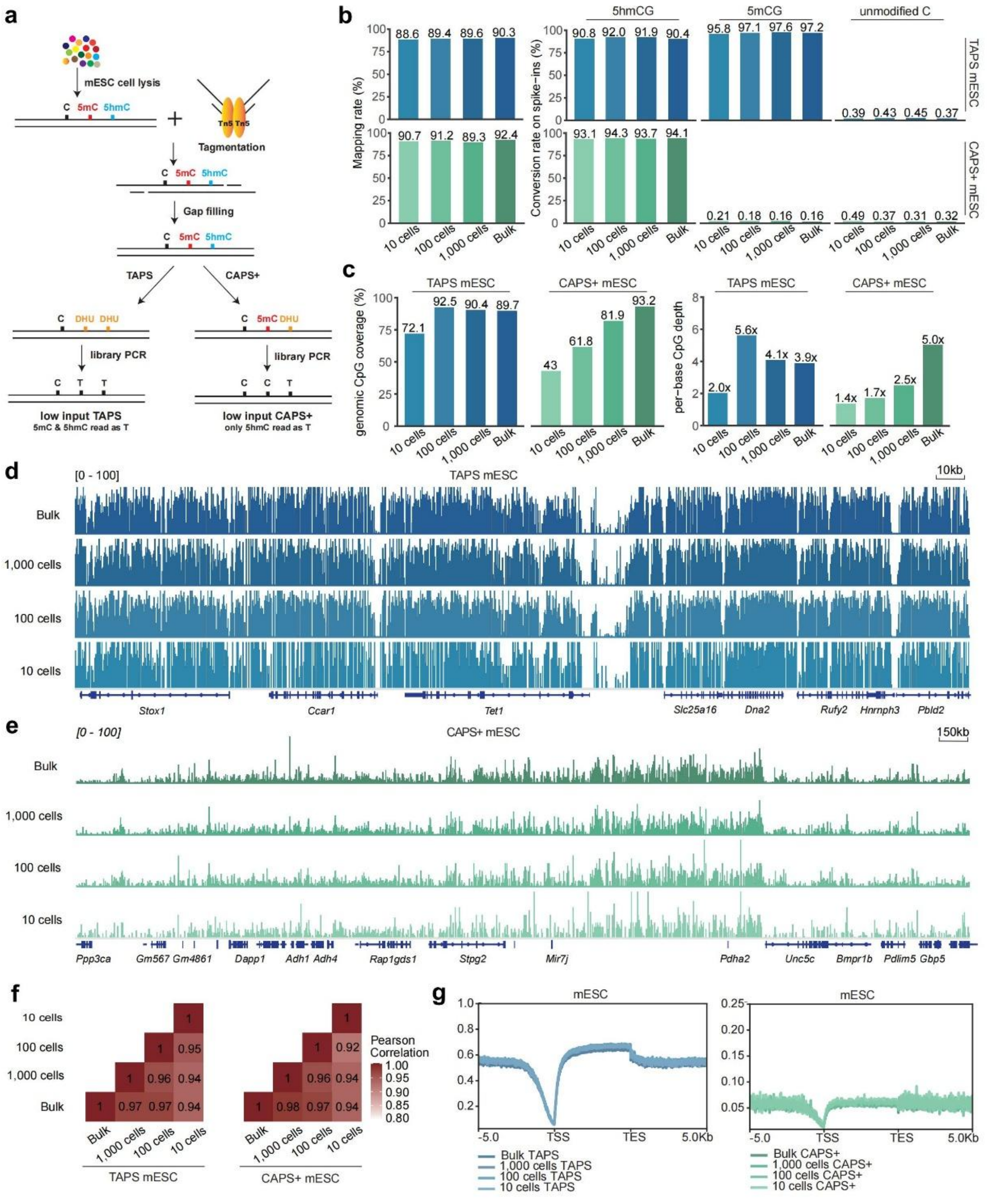
a
mESC cell lysis
Tagmentation
Gap filling
TAPS
CAPS+
library PCR
low input TAPS
5mC & 5hmC read as T
low input CAPS+
only 5hmC read as T
b
Mapping rate (%)
Conversion rate on spike-ins (%)
5hmCG
5mCG
unmodified C
TAPS mESC
CAPS+ mESC
10 cells
100 cells
1,000 cells
Bulk
c
genomic CpG coverage (%)
per-base CpG depth
d
TAPS mESC
e
CAPS+ mESC
f
Pearson Correlation
g
mESC
TSS
TES
Bulk TAPS
1,000 cells TAPS
100 cells TAPS
10 cells TAPS
Bulk CAPS+
1,000 cells CAPS+
100 cells CAPS+
10 cells CAPS+

Fig. S1: Developing low input TAPS and CAPS+ in mESC

a. Overview of the low input TAPS and CAPS+ methodologies. **b**. Barplots showing mapping rates (left), conversion rates based on 5hmCG, 5mCG, and false positives on unmodified C spike-ins (right). The values above represent the means. Top: TAPS. Bottom: CAPS+. **c**. Barplots showing genomic CpG coverage (left), and per-base CpG depth (right). **d, e.** IGV tracks showing 5mCG level along chr10:62,120,000-62,520,000 in TAPS (top) and 5hmCG level along chr3: 136,494,086-142,494,086 in CAPS+ (bottom). **f.** Heatmap showing Pearson correlation coefficient between bulk and low input TAPS/CAPS+. **g**. Metagene plots showing 5mCG and 5hmCG distribution along gene body from 5kb upstream of TSS (Transcription Start Sites) to 5kb downstream of TES (Transcription End Sites).

Fig. S2.

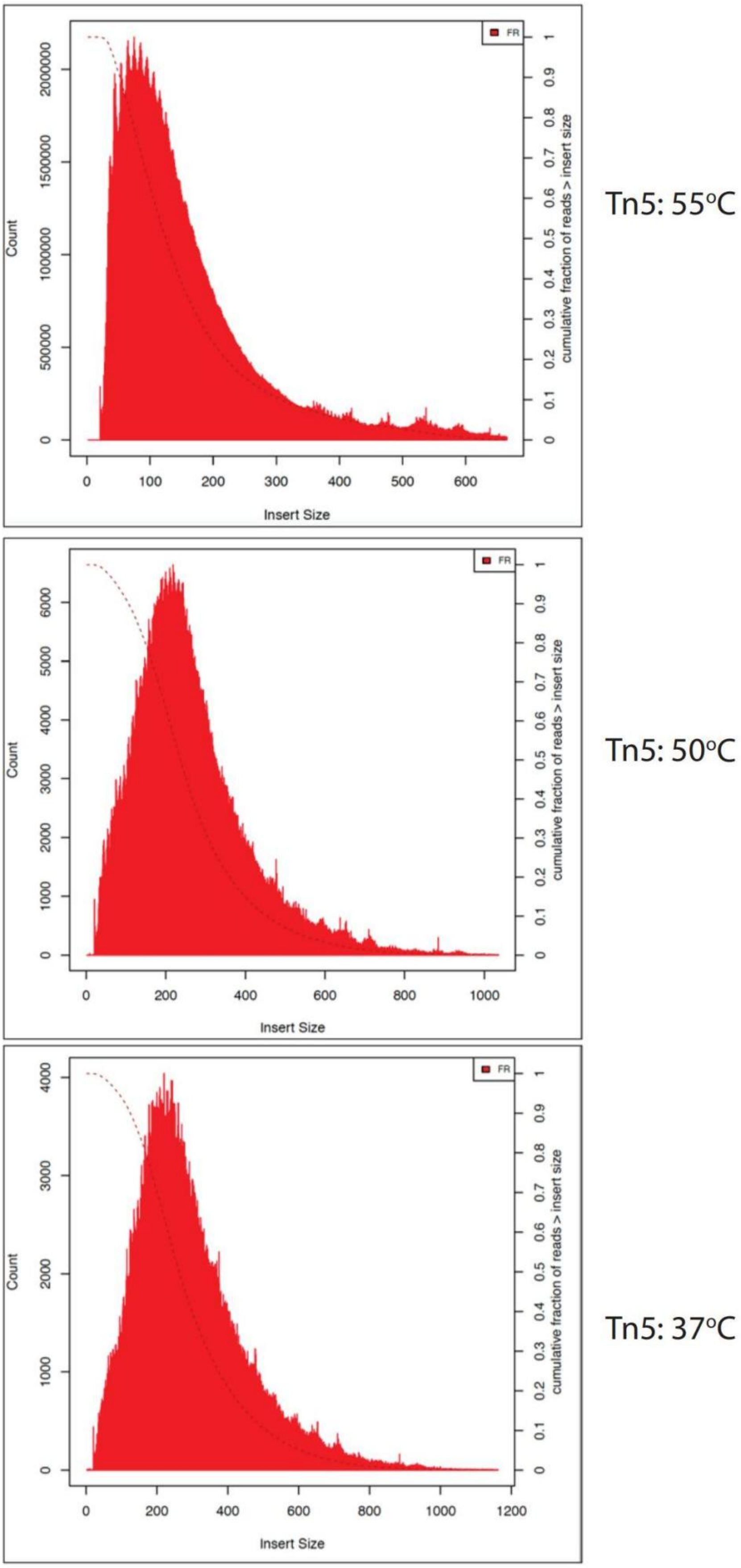
Tn5: 55°C
Tn5: 50°C
Tn5: 37°C
Count
Insert Size
cumulative fraction of reads > insert size
FR

Fig. S2. The library insert size distribution

The library inset size distribution by sequencing corresponds to different Tn5 working temperature: 55℃, 50℃, 37℃, respectively.

Fig. S3

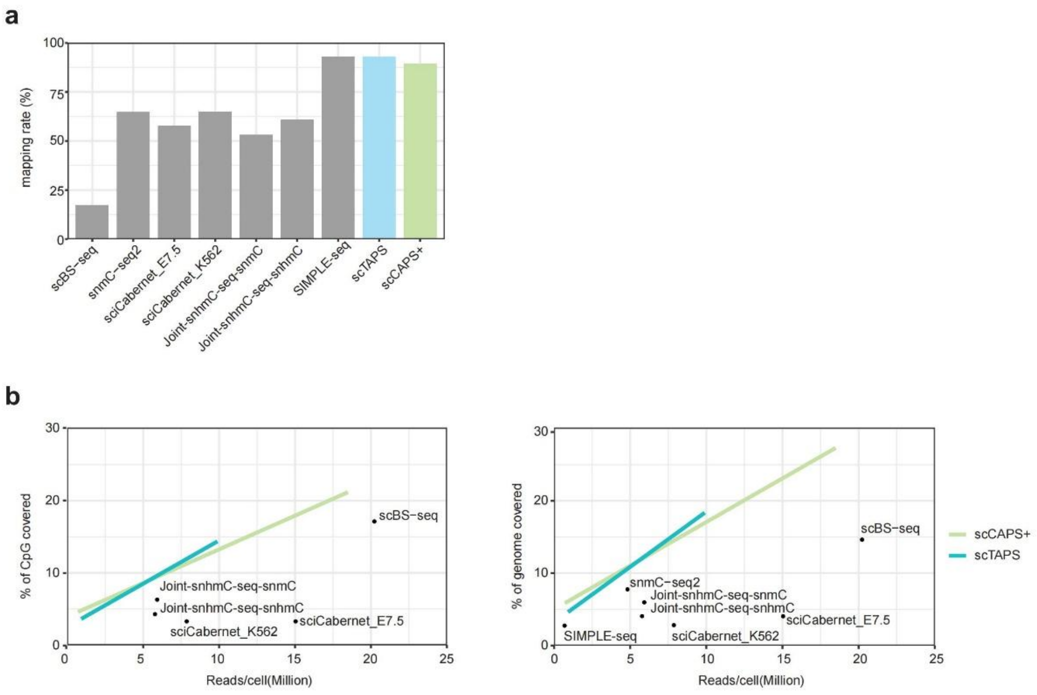

Fig. S3. Comparison of scTAPS/CAPS+ with previous single-cell sequencing techniques

a. Bar plots illustrating the mapping rates of scTAPS/CAPS+ in comparison with snmC-seq2, scBS-seq, SIMPLE-seq, sci-Cabernet, and Joint-snhmC-seq.

b. The line plot showing the percentage of covered CpG (left) and genome (right) at different sequencing reads per cell for scTAPS in CD8+ T cell and scCAPS+ in mESC. The dots represent average CpG coverage (left) or genome coverage (right) and average sequenced reads for each cell in snmC-seq2, scBS-seq, SIMPLE-seq, sci-Cabernet, and Joint-snhmC-seq.

Fig. S4

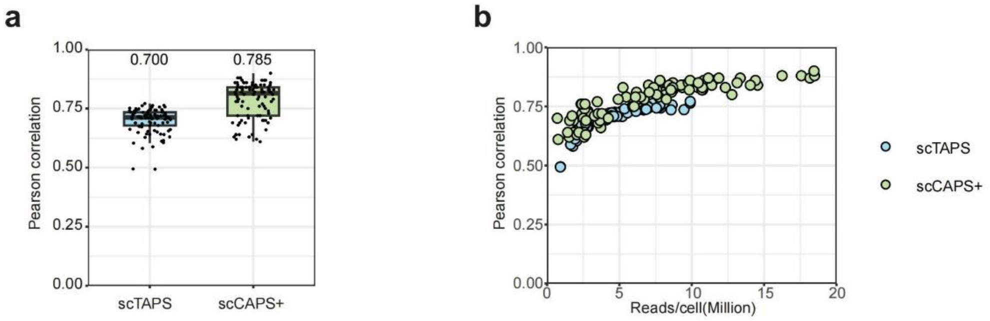

Fig. S4. Correlation analysis of methylation between individual cell and bulk 5mC/5hmC profiles

a. Box plot demonstrating the Pearson correlation of methylation between individual cells and bulk data for both scTAPS in CD8+ T cell and scCAPS+ in mESC.

b. The scatter plot showing the Pearson correlation of methylation between each cell and bulk at corresponding sequencing depth per cell for both scTAPS and scCAPS+.

Fig. S5

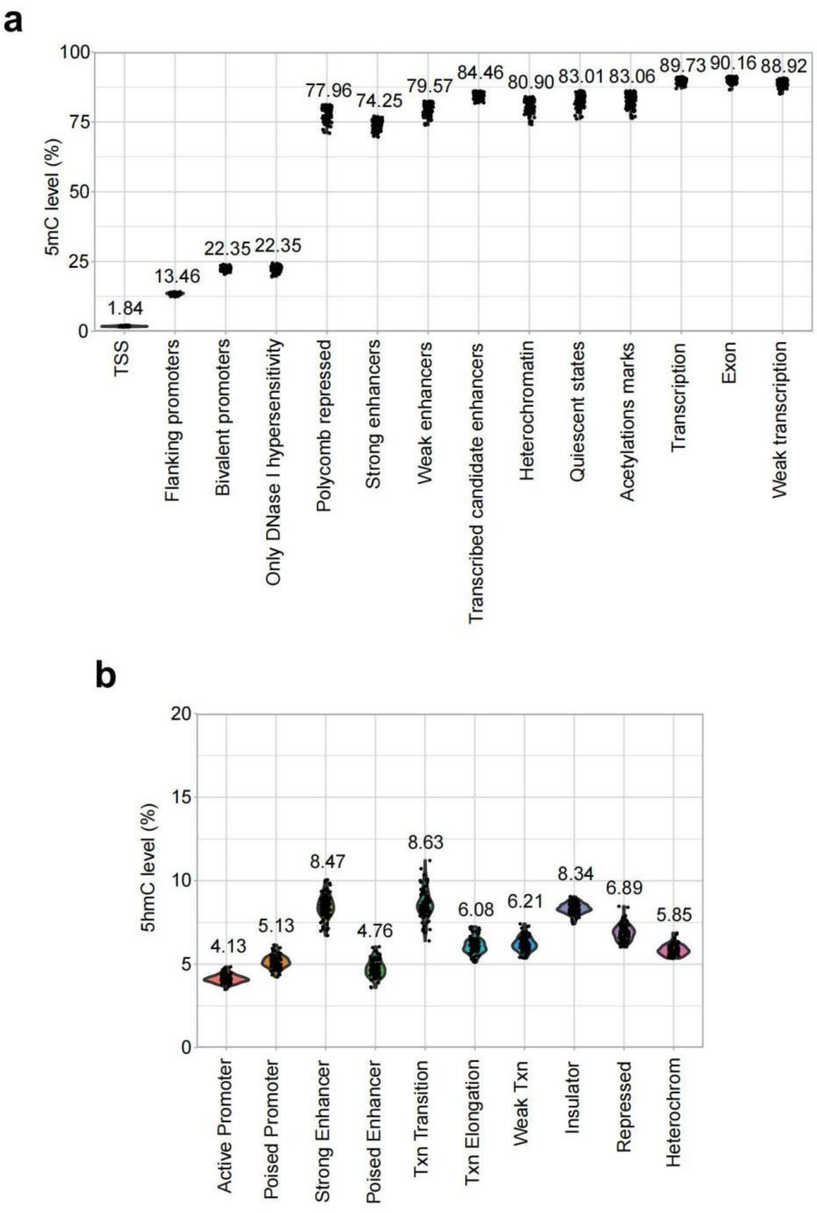

Fig. S5. 5mC and 5hmC levels at different genomic elements

Aggregated single cells' 5mC levels in CD8+ T cells (a) and 5hmC levels in mESC (b) around different genomic regions detected by scTAPS/scCAPS+

Fig. S6

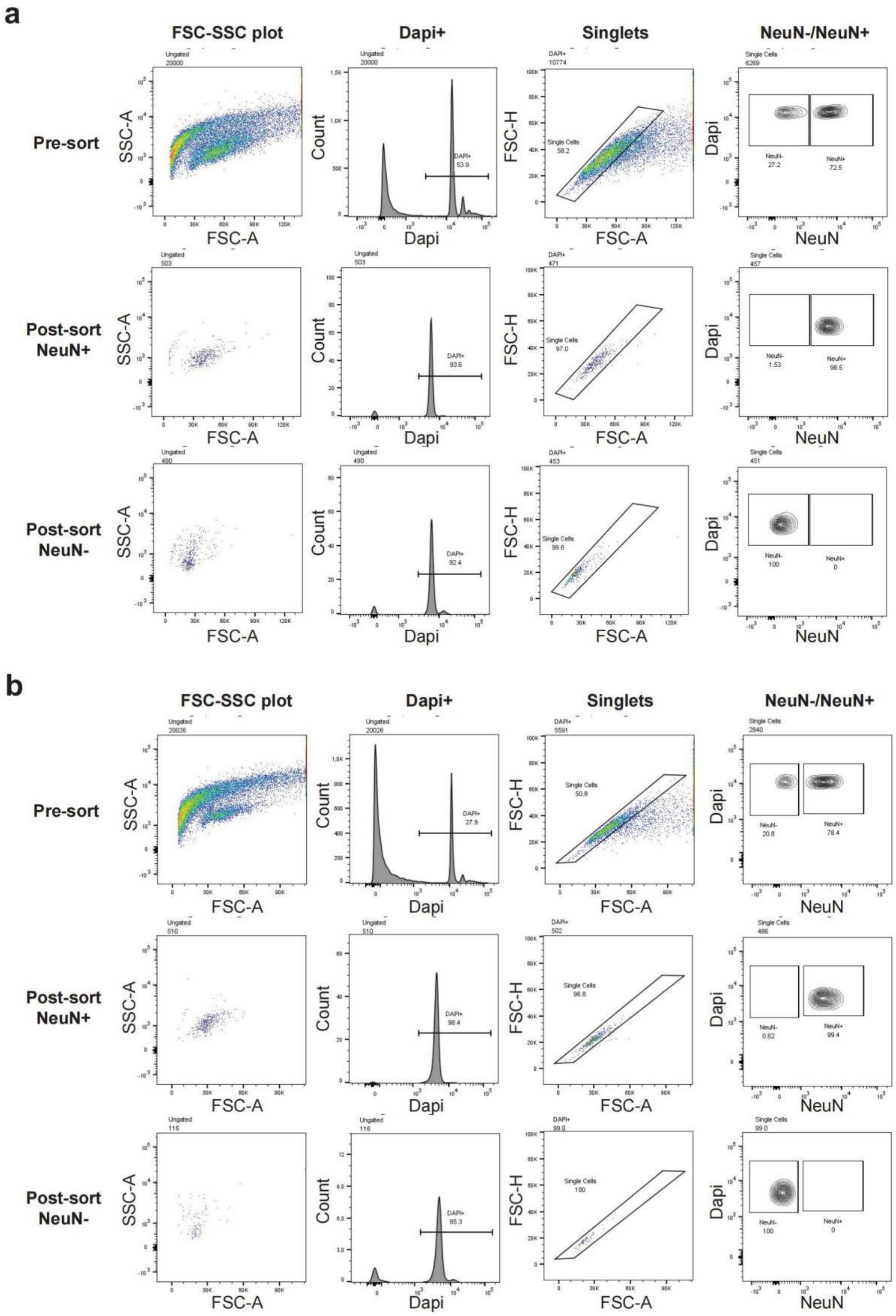
a
FSC-SSC plot
Dapi+
Singlets
NeuN-/NeuN+
Pre-sort
Post-sort NeuN+
Post-sort NeuN-
SSC-A
FSC-A
Count
Dapi
FSC-H
NeuN
b
FSC-SSC plot
Dapi+
Singlets
NeuN-/NeuN+
Pre-sort
Post-sort NeuN+
Post-sort NeuN-
SSC-A
FSC-A
Count
Dapi
FSC-H
NeuN

Fig. S6. Separation of young and aged mice neurons (NeuN+) and non-neurons (NeuN-) by FACS

a, b. Nuclei isolated from hippocampal tissues of 3m (a) and 18m (b) mice were stained with the anti-NeuN antibody and sorted by FACS. Single nuclei were captured by gating on DAPI-positive events, and then gating on Alexa Fluor 488 (NeuN) signal. Plots showing the pre-sorted and post-sorted NeuN+/NeuN- nuclei were listed.

Fig. S7

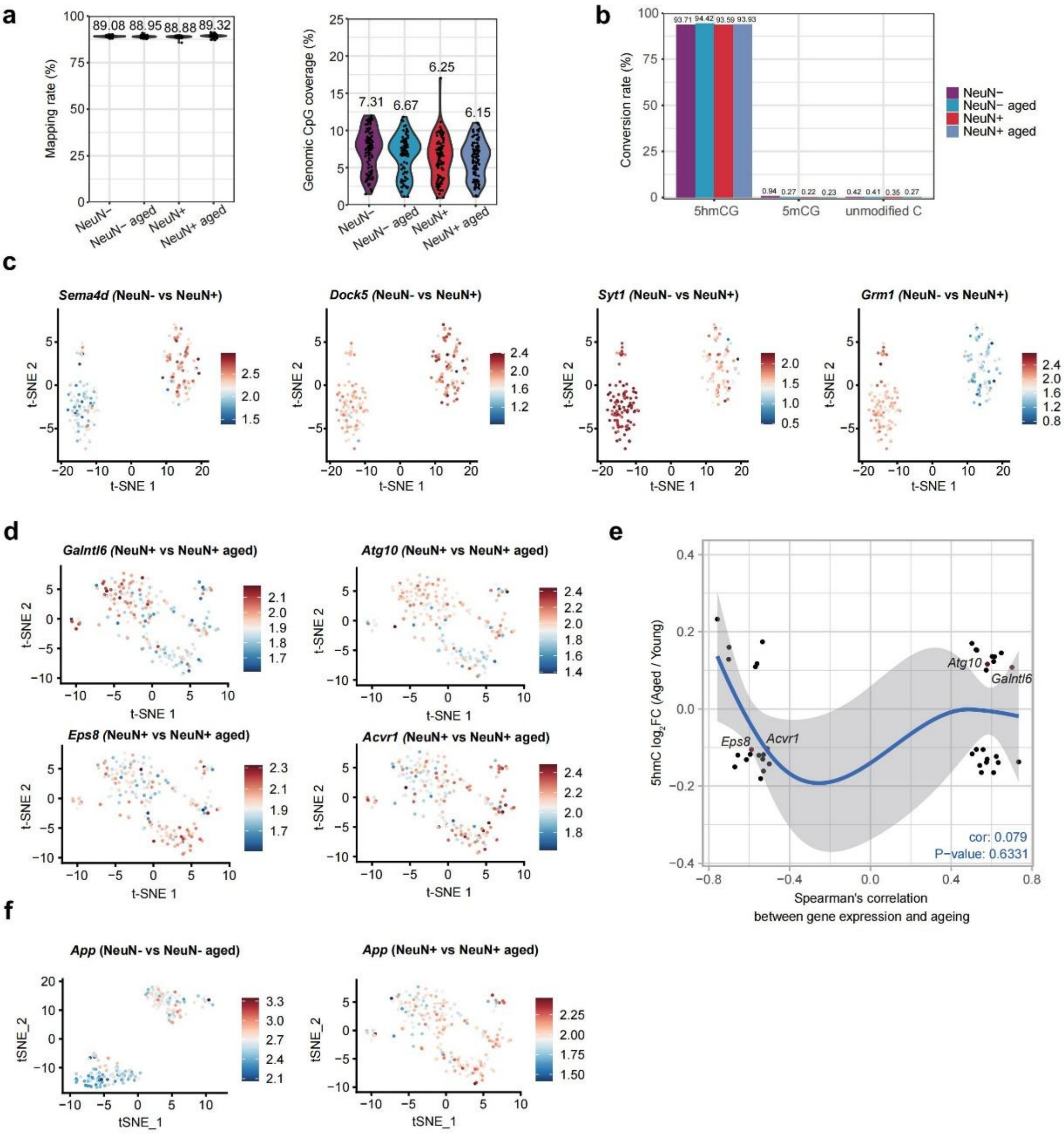

Fig. S7. scCAPS+ sequencing results in hippocampus during aging

a. Violin plots showing the mapping rates and genomic CpG coverage of scCAPS+ in mouse hippocampus, each point represents a single cell. The means are shown above. **b**. Conversion rate and false positive of scCAPS+ in mouse hippocampus (96 cells for each type). **c.** t-SNE plot showing 5hmC levels of non-neurons marker genes *Sema4d*

and *Dock5* and neurons marker genes *Syt1* and *Grm1.* **d.** t-SNE plot showing 5hmC levels of *Galntl6* and *Atg10, Eps8 and Acvr1*, in young and aged neurons. **e.** The scatter plot showing the 5hmC changes in neurons during aging, along with the corresponding Spearman's correlation coefficient depicting the relationship between gene expression and aging in the mouse brain. Pearson correlation coefficient and significance level were computed. **f**. t-SNE plot showing 5hmC levels of *App* gene in young and aged hippocampus (non-neurons and neurons).
