# Supplementary material for: Direct and bisulfite-free 5-methylcytosine and 5-hydroxymethylcytosine sequencing at single-cell resolution with scTAPS and scCAPS +
Source: Genome Biol. 2025 Aug 18;26:244. doi: 10.1186/s13059-025-03708-1 (PMC12359873; doi:10.1186/s13059-025-03708-1)
Supplement: Supplementary file 6 — Additional file 6: Review history [file 13059_2025_3708_MOESM6_ESM.odt]

1st round
Reviewer 1
The manuscript, "Direct, bisulfite-free 5-methylcytosine and 5-hydroxymethylcytosine sequencing at single-cell resolution" by Chen et al., developed a single-cell version of TAPS and CAPS+ to quantify the 5mC+5hmC and 5hmC at single base resolution. They first validated the data quality of scTAPS in CD8+ T cells and scCAPS+ in mESCs, respectively. Compared with bisulfite-based single-cell methods, scTAPS and scCAPS+ exhibited high mapping ratio and low false positive rate. They also investigated 5hmC dynamics during aging in the mouse hippocampus. These advanced techniques will significantly enhance DNA modification research, particularly in rare and valuable biological samples. Below are points of criticism.

Major point.
1. One of the advantages of scTAPS and scCAPS+ is the high mapping rate and CpG coverage. In Fig 1b, compared to other methods at single-base and single-cell resolution, how's the CpG coverage and genomic coverage of scTAPS and scCAPS+ under comparable sequencing depth? Such as snmC-seq2 (Luo et al., Nat Commun., 2018), scBS-seq (Clark et al., Nat Protoc., 2017), SIMPLE-seq (Bai et al., Nat Biotechnol., 2024), sci-Cabernet (Cao et al., PNAS., 2023), and Joint-snhmC-seq (Fabyanic et al., Nat Biotechnol.,2023).
2. n Figure 2h and lines 106-108, regarding the clustering analysis of 5hmC in the mouse hippocampus, what bin size was used to calculate the 5hmC content? Additionally, which factor is more dominant for cell clustering based on 5hmC signals: cell type or age? An integrated analysis of both neurons and non-neuronal cells in adult and aged mice using the same tSNE plot could provide a clearer perspective. Besides 5hmC on CpG sites, what is the modification level on CH sites in the hippocampus?
3. The single-cell resolution of scCAPS+ allows for the exploration of cellular heterogeneity. Regarding 5hmC signals, are there any genes with stable and dynamic 5hmC modifications during aging?

Minor point.
1. In figure S1, what's the material for 10-cell, 100-cell, 1000-cell and bulk level of TAPS and CAPS+, respectively? It would be clearer if the sample source is denoted in the figure or figure legend.
2. Punctuation typos. In line 180, it should be "(RosetteSep, StemCell Technologies, #15023).". In line 285, it should be "…as described before[24] and their sequences ".
3. In Additional file 1 and 2, what did the "rC", "aC", "nC" and "mC" stand for? And the legends of Additional files were missing.

Reviewer 2
In the manuscript entitled "Direct, bisulfite-free 5-methylcytosine and 5-hydroxymethylcytosine sequencing at single-cell resolution" the authors provide two new, bisulfite free scDNA methylation methods. The authors build upon their past bulk-based methods TET-assisted pyridine borane sequencing (TAPS) and chemical-assisted pyridine borane sequencing plus (CAPS+) for direct 5mC and 5hmC detection, and adapted them for single-cell approaches. In this way, the authors provide two interesting plate-based low-throughput approaches for scDNA methylation, that are very relevant for the study of for example rare cell types, where no high cell numbers can be obtained and high-throughput methods can thus not be applied. 

Major comments

1. The method used spike-in controls. This is an elegant way to check the conversion rates. I suggest stating this in the text, so the reader can appreciate this here and does not have to go to the methods.

2. The correlation between 3 individual cells and the bulk is given in figure 1d. Regarding this, it would be good to state in the text for each experiment how many cells in total were analysed. And for this specific figure to plot also the correlation of each cell versus the bulk in a boxplot/violin plot to appreciate the variation. 

3. It is unclear how marker genes were defined to 5hmC levels. It is clear that the authors define genes with differential 5hmC levels between the Neuron and Non-Neuron population. But then, if the 5hmC is higher let's say in population 1, does the marker gene belong to this population? And if so, what is the biological reasoning behind? Later on the authors say that 5hmC levels are higher in the marker genes for the given population. If they come to this conclusion later on, they cannot have used the 5hmC levels in the first place to assign the marker genes. From here, I assume that the authors blindly defined two populations with two sets of marker genes and then automatically assigned them to Non-Neurons based on the prior knowledge they have of the populations. Or maybe they used a different approach. The authors should clearly state how this was done. And, the biological reasoning behind.

4. In the comparison of the method with SAMPLE-seq it would be fair to compare the genome coverage based on an equal number of mapped reads per cell. Equally the authors should state why the SAMPLE-seq method uses a standard curve for modification level validation, while TAPS and CAPS+ do not need that. 

5. Methods:
-The source of human blood cells is not clear, neither if informed consent was obtained.
-The authors state to have a sequencing depth of 4.8 and 7.7 million reads per single cell in the text, but in the methods it is stated that cells with an excessively high count (>3,000,000) were systematically excluded from downstream analysis. This seems counterintuitive, how can one have 4.8 and 7.7 million reads on average while all cells with >3M mapped reads are removed. This is maybe due to the fact that 4.8-7.7M are total reads and 3M are mapped reads. But mapping rates are really high so the difference cannot be huge. The authors should explain this and adjust the text (adjusting the numbers and/or stating it is total or mapped reads) and/or the methods to clarify this.

Minor comments

6. I suggest to amend the abstract saying that this is a plate-based method, this will help the reader to understand how it can be implemented as well as its throughput.

7. Figures:
-In figure 2b and 2h, I suggest to plot only the right panels. They are informative and convincing to see the separation of the cells by cell type/age.
-In figures 2f and 2i and their related supplements, I suggest to always put in the title which cells are analysed, e.g in 2f: Neurons vs Non-neurons, or in 2i: Aged vs Non-aged Non-neurons. Otherwise, the reader has to check every time the shape of the UMAP or the legend. 

8. I think in page 7 the word "Conversation" is a typo, probably needs to be "Conversion".

Reviewer 3
Chen et al. have adapted the Tn5 transposase-mediated genome fragmentation technique, which has been widely used in various single-cell and low-input sequencing methods, to the TAPS and CAPS+ for sequencing 5mC and 5hmC at low-input and single-cell levels. They first validated the techniques by showing a high correlation between bulk population sequencing and single-cell sequencing data of CD8+ T cells. Next, they performed scTAPS and scCAPS+ sequencing analyses on neuronal and non-neuronal cells in the mouse hippocampus at the single-cell level, revealing that the overall hmC levels in both cell types increased with age. Although the novelty of these methods is somewhat limited and the conclusions drawn from the analysis do not necessarily require single-cell resolution, the technique still holds considerable application value. Below are several issues to be addressed:
1. Single-cell sequencing is expected to facilitate a further classification of cell types based on epigenetic modifications, but this work did not leverage this technique for such analysis. Given that the NeuN-sorted cell populations already represent distinct cell types, it is not surprising that hmC can distinguish these groups.
2. Line 121: "Conversation only happens on 5mC and 5hmC which will preserve the genomic complexity, thus leading to low false positives and high sequencing quality." This statement is not entirely accurate because unmodified C (in TAPS and CAPS+) and mC (in CAPS+) can also undergo conversion (Fig 1b, S1b). Given that unmodified C constitutes the vast majority of the genome, even a very low conversion rate could result in a significant number of false-positive signals. Additionally, did you mean "conversation" or "conversion"?
3. Is the global hmC quantification in neuronal and non-neuronal cells of the hippocampus by mass spectrometry consistent with the levels obtained from scCAPS+ sequencing (Fig 2a)?
4. In Fig 1b and Fig S5b, the Y-axis labeling should be changed to "conversion rate." Additionally, the legend should specify the number of single cells used.
5. In Fig 2f, what does the scale bar represent?
6. In Fig S1b, how were the spike-ins adjusted for different cell input amounts?

Reviewer 4
The manuscript presents direct sequencing methodologies, scTAPS and scCAPS+, designed for the quantitative detection of 5mC and 5hmC at single-base resolution in single cells. These methods achieve high mapping efficiencies and demonstrate accurate profiling of 5mC and 5hmC in CD8+ T cells and mES cells. A notable finding is the global increase in 5hmC within the hippocampus of aging mice, observed in both neurons and non-neurons. Overall, the manuscript makes a significant contribution to the field of single-cell epigenomics by introducing new tools for the direct detection of 5mC and 5hmC. 
A few comments: 
1. The authors need to cite previously published methods, such as 6-letter sequencing (PMID: 36747096) and SIMPLE-seq (PMID: 38336903), which allow for the simultaneous analysis of 5mC and 5hmC at both bulk and single-cell levels, in the Introduction as well as the Discussion of this article.
2. The text and legend associated with Figure 1e mention the levels of 5mCG and 5hmCG along a 7 MB region of chr 2 in human CD8+ T cells (top) and a 117 MB region of Chr 12 in mouse ES cells but only provide data for one of the two modifications in each case. The authors should provide both 5mCG and 5hmCG data for both T cells and mouse ES cells. Also, it would be worthwhile to show a similar-sized (10-30 MB) region for the mouse ES cells, for instance the region currently at the very right of the mESC panel, since there is a greater range of 5hmC levels in this region.
3. In Figures 1d-f, the authors compare bulk and single-cell data, the results show little difference between the two. Can the authors strengthen the analysis by highlighting any unique insights provided by single-cell sequencing that are not apparent from bulk sequencing?
4. In Figure 2a, the authors observed a global increase in 5hmC within the hippocampus of aging mice, evident in both neurons and non-neurons. What is the expression status of TET1, TET2, and TET3 in these cells?
5. The authors only have single-cell sequencing data for Figure 2, unlike Figure 1 which includes bulk sequencing data. It would be useful if the authors could supplement Figure 2 with bulk sequencing data to compare bulk and single-cell data and determine if there are specific findings in the single-cell data.
6. Although the manuscript briefly compares scTAPS/scCAPS+ with SIMPLE-seq methods, a more detailed comparison of their bulk RNA-seq data across a wider range of methodologies including 6-letter sequencing and SIMPLE-seq techniques would provide a clearer picture of the advantages and potential limitations of the new methods.

Minor
1. The author profiles 5mC and 5hmC in CD8+ T cells and mouse ES cells in Figure 1. However, Figures 1b-f do not indicate whether the analyzed data is from T cells, mouse ES cells, or both cell types combined. Can the authors provide specific clarification?
2. Line 124: It should be 'SIMPLE-seq,' not 'SAMPLE-seq.

Authors’ response
Reviewer #1: The manuscript, "Direct, bisulfite-free 5-methylcytosine and 5-hydroxymethylcytosine sequencing at single-cell resolution" by Chen et al., developed a single-cell version of TAPS and CAPS+ to quantify the 5mC+5hmC and 5hmC at single base resolution. They first validated the data quality of scTAPS in CD8+ T cells and scCAPS+ in mESCs, respectively. Compared with bisulfite-based single-cell methods, scTAPS and scCAPS+ exhibited high mapping ratio and low false positive rate. They also investigated 5hmC dynamics during aging in the mouse hippocampus. These advanced techniques will significantly enhance DNA modification research, particularly in rare and valuable biological samples. Below are points of criticism.

Response： We are glad to hear the reviewer’s recognition of the advance of our study and appreciate the constructive comments. Please find the point-to-point response below.

Major point.
1.  One of the advantages of scTAPS and scCAPS+ is the high mapping rate and CpG coverage. In Fig 1b, compared to other methods at single-base and single-cell resolution, how's the CpG coverage and genomic coverage of scTAPS and scCAPS+ under comparable sequencing depth? Such as snmC-seq2 (Luo et al., Nat Commun., 2018), scBS-seq (Clark et al., Nat Protoc., 2017), SIMPLE-seq (Bai et al., Nat Biotechnol., 2024), sci-Cabernet (Cao et al., PNAS., 2023), and Joint-snhmC-seq (Fabyanic et al., Nat Biotechnol.,2023).

Response: To address the reviewer's concern, we compared different methods and have plotted the mapping rate (new supplemental figure, Fig. S3a), the relationship between the number of raw sequencing reads per cell and the CpG/genome coverage per cell (new supplemental figure, Fig. S3b). The results demonstrate that scTAPS and scCAPS+ achieve higher mapping rates compared to indirect methods, and higher CpG and genomic coverage compared to snmC-seq2 (Luo C, et al., Nat Commun. 2018. PMID: 30237449), scBS-seq (Smallwood SA, et al., Nat Methods. 2014. PMID: 25042786), sci-Cabernet (Cao Y, et al., PNAS. 2023. PMID: 38011566), and Joint-snhmC-seq (Fabyanic EB, et al., Nat Biotechnol. 2024. PMID: 37640946) when assessed under comparable sequencing depths.

New Fig. S3a
A graph of a number of different types of numbers

AI-generated content may be incorrect.
New Fig. S3b
A graph of a function

AI-generated content may be incorrect.
2. In Figure 2h and lines 106-108, regarding the clustering analysis of 5hmC in the mouse hippocampus, what bin size was used to calculate the 5hmC content? Additionally, which factor is more dominant for cell clustering based on 5hmC signals: cell type or age? An integrated analysis of both neurons and non-neuronal cells in adult and aged mice using the same tSNE plot could provide a clearer perspective. Besides 5hmC on CpG sites, what is the modification level on CH sites in the hippocampus?

Response: In the clustering analysis of 5hmC in the mouse hippocampus, 5hmC levels were calculated based on each annotated protein-coding gene. We also visualized both neurons and non-neuronal cells from young and aged mice within a single plot (Data #1) as suggested, and we found cell type emerged as the dominant factor influencing cell clustering based on 5hmC signals.

Data #1 to reviewer only
A group of graphs showing different colored dots

AI-generated content may be incorrect.
We observed low levels of hydroxymethylation (5hmC) at CH sites, with 0.36% in non-neurons and 0.51% in neurons, which is only slightly higher than the false-positive rate observed (0.18 to 0.20% at CH sites in unmodified 2kb regions). This pattern of low 5hmC at CH sites is consistent with findings from Joint-seq (Fabyanic EB, et al., Nat Biotechnol. 2024, PMID: 37640946), which reported 0.23% for non-neurons and 0.39% for neurons, with a false-positive rate of 0.26%. Given the challenges in reliably detecting 5hmC at such low levels in the CH context, we focused our downstream analysis on 5hmC levels at CG contexts.


3.  The single-cell resolution of scCAPS+ allows for the exploration of cellular heterogeneity. Regarding 5hmC signals, are there any genes with stable and dynamic 5hmC modifications during aging?

Response: We appreciate the reviewer’s comments. In response, we have provided a detailed list of gene-body 5hmCG levels for all protein-coding genes (new Additional File 2, Table 5). For the dynamic 5hmC modification, we identified specific genes with notable changes in 5hmC levels during the aging process, such as Galntl6, Atg10, Eps8 and Acvr1 as shown in figure S7d and Additional file 2 (as well as in Data #2). Conversely, we also found a subset of genes, including Sipa1l1, Slc9a9, Jazf1 and Pip5k1b, some of which also play vital roles in neurons, exhibited consistently stable 5hmC modifications across during aging (Data #2). As for the cellular heterogeneity, we observed slightly lower variation for Atg10 gene in aged neurons compared to young neurons. This might be a technical artifact due to the sparsity of single-cell data and the limited number of cells analyzed, but it could also suggest that Atg10 may play a more regulated role in aged neurons, which require stable maintenance of its 5hmC level.

Data #2 to reviewer only
A screenshot of a graph

AI-generated content may be incorrect.
Minor point.
1.  In figure S1, what's the material for 10-cell, 100-cell, 1000-cell and bulk level of TAPS and CAPS+, respectively? It would be clearer if the sample source is denoted in the figure or figure legend.

Response: We appreciate the reviewer’s comment. The low-input samples (10, 100, and 1000 cells) are derived from mouse embryonic stem cells (mESC). We have now updated both figure S1 and the corresponding figure legends to clearly indicate this information.

2. Punctuation typos. In line 180, it should be "(RosetteSep, StemCell Technologies, #15023).". In line 285, it should be "…as described before [24] and their sequences ".

Response: We appreciate the reviewer’s comments, and the punctuation typos have been corrected as follows:
In lines 161-162: 
Human CD8+ T cells were isolated from blood cones using negative immunodensity selection with CD8+ (RosetteSep, StemCell Technologies, #15023).
In lines 256-257: 
96 combinatory Tn5 assembly were performed as described before [27], and their sequences are presented herein. 

3. In Additional file 1 and 2, what did the "rC", "aC", "nC" and "mC" stand for? And the legends of Additional files were missing.

Response: We appreciate the reviewer’s comments. The abbreviations are defined as follows: "rC" represents the methylation ratio; "aC" indicates the total cytosine; "mC" refers to methylated cytosine; and "nC" denotes the number of cytosine sites. To improve clarity, we have updated the column names in Additional files 1 and 2. Additionally, we have included legends for additional files as suggested (line 639-668)


Reviewer #2: In the manuscript entitled "Direct, bisulfite-free 5-methylcytosine and 5-hydroxymethylcytosine sequencing at single-cell resolution" the authors provide two new, bisulfite free scDNA methylation methods. The authors build upon their past bulk-based methods TET-assisted pyridine borane sequencing (TAPS) and chemical-assisted pyridine borane sequencing plus (CAPS+) for direct 5mC and 5hmC detection and adapted them for single-cell approaches. In this way, the authors provide two interesting plate-based low-throughput approaches for scDNA methylation, that are very relevant for the study of for example rare cell types, where no high cell numbers can be obtained and high-throughput methods can thus not be applied.

Response: We appreciate the reviewer’s positive comments on our current plate-based  scTAPS/scCAPS+ for single-cell direct 5mC and 5hmC detection. We anticipate this Tn5 based method can easily be adopted to high throughput when huge cell number is needed. Please find the point-to-point response below.

Major comments
1. The method used spike-in controls. This is an elegant way to check the conversion rates. I suggest stating this in the text, so the reader can appreciate this here and does not have to go to the methods.

Response: We appreciate the reviewer's valuable suggestion. We have now explicitly stated the use of spike-in controls to assess conversion rates in the manuscript. 
In lines 77-80:
Notably, using incorporated spike-in controls, we observed robust conversion rates in both scTAPS (5mCG: 96.6%, 5hmCG: 85.0%) and scCAPS+ (5hmCG: 93.0%), along with very low false positive rates in scTAPS (uC: 0.19%) and scCAPS+ (uC: 0.38%, 5mCG: 0.25%) (Fig 1b).

2. The correlation between 3 individual cells and the bulk is given in figure 1d. Regarding this, it would be good to state in the text for each experiment how many cells in total were analysed.  And for this specific figure to plot also the correlation of each cell versus the bulk in a boxplot/violin plot to appreciate the variation. 

Response: We appreciate the reviewer's valuable suggestion. We have now explicitly mentioned in the text that a total of 96 CD8+ T cells were analyzed for scTAPS, and 96 mESC  were analyzed for scCAPS+, respectively. Additionally, we have included a new box plot illustrating the Pearson correlation of methylation between each cell and bulk for both scTAPS and scCAPS+ (new supplemental figure, Fig. S4a, b). The average Pearson correlation is 0.700 for scTAPS and 0.785 for scCAPS+, which can be further improved by increased sequencing depth.

New Fig. S4a, b
A graph with a diagram and a graph with a diagram

AI-generated content may be incorrect.
3. It is unclear how marker genes were defined to 5hmC levels. It is clear that the authors define genes with differential 5hmC levels between the Neuron and Non-Neuron population. But then, if the 5hmC is higher let's say in population 1, does the marker gene belong to this population? And if so, what is the biological reasoning behind? Later on the authors say that 5hmC levels are higher in the marker genes for the given population. If they come to this conclusion later on, they cannot have used the 5hmC levels in the first place to assign the marker genes. From here, I assume that the authors blindly defined two populations with two sets of marker genes and then automatically assigned them to Non-Neurons based on the prior knowledge they have of the populations. Or maybe they used a different approach. The authors should clearly state how this was done. And, the biological reasoning behind.

Response: We appreciate the reviewer’s comments and apologize for the confusion. To conduct the clustering analysis, we calculated the 5hmCG levels across the gene body for each gene. Using these data as input, we performed an unbiased clustering using the FindClusters function in Seurat. As shown in Figure 2b, the clusters we obtained align well with the cell types identified by FACS: population 1 corresponds to NeuN- cells, and population 2 corresponds to NeuN+ cells. To determine markers for NeuN+ and NeuN- cells, we employed the FindMarkers function in Seurat. This analysis identified genes with differential 5hmC levels between the two populations. The avg_log2FC value reflects the log fold-change of the average 5hmC levels between the groups: a positive value indicates a higher 5hmC modification level in group 1 (NeuN-), while a negative value indicates a higher 5hmC modification level in group 2 (NeuN+).
We have added more details in the method section for clarification (Line 363-367)


4. In the comparison of the method with SAMPLE-seq it would be fair to compare the genome coverage based on an equal number of mapped reads per cell. Equally the authors should state why the SAMPLE-seq method uses a standard curve for modification level validation, while TAPS and CAPS+ do not need that.

Response: To address the reviewer's concern, we have plotted the correlation between the number of raw sequencing reads per cell and the CpG/genome coverage per cell (new supplemental figure, Fig. S3b). The results demonstrate that scTAPS and scCAPS+ achieve higher CpG and genomic coverage compared to not only SIMPLE-seq, but also the other methods, like snmC-seq2 (Luo C, et al., Nat Commun. 2018. PMID: 30237449), scBS-seq (Smallwood SA, et al., Nat Methods. 2014. PMID: 25042786), sci-Cabernet (Cao Y, et al., PNAS. 2023. PMID: 38011566), and Joint-snhmC-seq (Fabyanic EB, et al., Nat Biotechnol. 2024. PMID: 37640946) when assessed under comparable sequencing depths. 

New Fig. S3b
A graph of a function

AI-generated content may be incorrect.
Additionally, we explicitly stated in the text that SIMPLE-seq requires adjustment using a standard curve due to its incomplete conversion rate for 5mC (approximately 87%). In contrast, our method achieves a conversion rate of 96.6%, which is nearly complete and does not require the same adjustment as SIMPLE-seq. 
In line 133-135:
However, compared to our method, SIMPLE-seq exhibited lower conversion rates (~87% for 5mC), necessitating the use of a standard curve for modification level adjustment. 

5.Methods:
-The source of human blood cells is not clear, neither if informed consent was obtained.

Response: We appreciate the reviewer’s comments and apologize for any lack of clarity. To address this concern, we have revised the relevant text to explicitly clarify the source of human blood cells and confirm that informed consent was obtained. Please see the updated statement:
In line 390-395:
This project was conducted under the non-clinical issue division of the National Health Service and the Inter-Divisional Research Ethics Committee of the University of Oxford (RECs 11/H0711/7, and Oxford Biobank Application number 18/A019; IRAS Project ID:66954). An anonymous healthy individual gave informed consent to use their blood by means of blood cones to extract lymphocytes for the experiments described.

-The authors state to have a sequencing depth of 4.8 and 7.7 million reads per single cell in the text, but in the methods it is stated that cells with an excessively high count (>3,000,000) were systematically excluded from downstream analysis. This seems counterintuitive, how can one have 4.8 and 7.7 million reads on average while all cells with >3M mapped reads are removed. This is maybe due to the fact that 4.8-7.7M are total reads and 3M are mapped reads. But mapping rates are really high so the difference cannot be huge. The authors should explain this and adjust the text (adjusting the numbers and/or stating it is total or mapped reads) and/or the methods to clarify this.

Response: We appreciate the reviewer’s comments, and we apologize for the confusion.  In Figure 1, the sequencing depths reached 4.8 million and 7.7 million reads per cell for scTAPS and scCAPS+ respectively, without excluding any cells at this stage. In Figure 2, we sequenced 6.5M reads per cell on average for scCAPS+ in mice hippocampus, and we excluded cells with over 3 million properly mapped reads, where properly mapped reads were defined using MAPQ >1 and Picard’s MarkDuplicates filtering. We have added more details in the method section for clarification (line 353-357). Our library exhibits a relatively high duplication rate (~74%), resulting in an average of 1.2 million properly mapped reads per cell. The threshold was set to exclude wells potentially containing multiple cells. This filtering approach aligns with methodologies used in other studies (Liu H, et al., Nature. 2021. PMID: 34616061; Luo C, et al., Science. 2017. PMID: 28798132). By applying this filtering, only one cell was excluded from further analysis.  

Minor comments
6. I suggest to amend the abstract saying that this is a plate-based method, this will help the reader to understand how it can be implemented as well as its throughput.

Response: We appreciate the reviewer’s comment. We have amended the abstract, accordingly, as noted in lines 31-33: ‘Achieving ~90% mapping efficiency, our plate-based methods accurately benchmark 5mC and 5hmC profiles in CD8+ T and mES cells.’ This will provide clarity regarding the implementation and throughput capabilities of the scTAPS/scCAPS+ techniques.

7. Figures:
-In figure 2b and 2h, I suggest to plot only the right panels. They are informative and convincing to see the separation of the cells by cell type/age.

Response: We thank the reviewer's suggestion. As the left panel shows the clustering assay results based on 5hmC modification, the right panel shows the FACS sorting results based on cell type/age. Although 5hmC modifications allow for clear separation of cell types (figure 2b) and age in non-neuronal cells (figure 2h, top), this separation is not as distinct in neurons (figure 2h, bottom). This indicates that the changes in 5hmC associated with aging in neurons are less pronounced compared to those in non-neuronal cells. The subtle differences observed between the left and right panels for neurons underscore this point. Therefore, we believe it is important to retain the left panel to illustrate the 5hmC separation difference of neurons and non-neuronal cells by age. 

-In figures 2f and 2i and their related supplements, I suggest to always put in the title which cells are analysed, e.g in 2f: Neurons vs Non-neurons, or in 2i: Aged vs Non-aged Non-neurons. Otherwise, the reader has to check every time the shape of the UMAP or the legend.

Response: We appreciate the reviewer's valuable suggestion. In response, we have now labeled the cell types in Figures 2f and 2i and related supplementary figures, to enhance clarity for the reader.

8. I think in page 7 the word "Conversation" is a typo, probably needs to be "Conversion".
Response: Thank you for pointing out the typo. It has been corrected to "Conversion".


Reviewer #3: Chen et al. have adapted the Tn5 transposase-mediated genome fragmentation technique, which has been widely used in various single-cell and low-input sequencing methods, to the TAPS and CAPS+ for sequencing 5mC and 5hmC at low-input and single-cell levels. They first validated the techniques by showing a high correlation between bulk population sequencing and single-cell sequencing data of CD8+ T cells. Next, they performed scTAPS and scCAPS+ sequencing analyses on neuronal and non-neuronal cells in the mouse hippocampus at the single-cell level, revealing that the overall hmC levels in both cell types increased with age. Although the novelty of these methods is somewhat limited and the conclusions drawn from the analysis do not necessarily require single-cell resolution, the technique still holds considerable application value. Below are several issues to be addressed:

Response: We appreciate the reviewer’s positive comments regarding our scTAPS/scCAPS+ for the detection of 5mC and 5hmC at the single-cell level. As this is primarily a methodological paper, our analysis focuses on ensuring sequencing quality and providing an overall evaluation of 5mC and 5hmC within simple biological processes. However, the current limitations related to throughput (cell numbers) also restrict our ability to conduct more detailed single-cell resolution analyses, such as accurate cell type clustering. We believe that our method has the potential to be readily adapted for high-throughput applications when a large number of cells is required. In the future, it holds significant promise for fulfilling the demands of single-cell resolution analysis. Please find our detailed point-by-point responses below.

1.  Single-cell sequencing is expected to facilitate a further classification of cell types based on epigenetic modifications, but this work did not leverage this technique for such analysis. Given that the NeuN-sorted cell populations already represent distinct cell types, it is not surprising that hmC can distinguish these groups.

Response: We appreciate the reviewer’s comment. To address the reviewer's concerns, we have tried to further cluster the neurons. Our analysis indicates the presence of two primary neuronal cell types: an excitatory neuron subtype (characterized by high hmCG  on Sv2b) and an inhibitory neuron subtype (characterized by high hmCG on Slc6a1) （Data #3）. Sv2b (Pazarlar BA, et al., Mol Brain. 2022. PMID: 35578248) and Slc6a1 (Mermer F, et al., Brain. 2021. PMID: 34028503) have been identified as marker genes of excitatory and inhibitory neurons, respectively, based on RNA expression profiles. This suggests that our methodology has the potential for subtype classification. This observation, however, was constrained by the limited number of cells analyzed. Furthermore, relying on single cell 5hmCG profiles alone is difficult to identify additional cell subtypes due to 5hmCG’s weak correlation with gene expression. Future studies could enhance the classification of neuronal cell subtypes by employing both scTAPS and scCAPS+ for 5hmC and 5mC profiles.

Data #3 to reviewer only
A screenshot of a graph

AI-generated content may be incorrect.

2.  Line 121: "Conversation only happens on 5mC and 5hmC which will preserve the genomic complexity, thus leading to low false positives and high sequencing quality." This statement is not entirely accurate because unmodified C (in TAPS and CAPS+) and mC (in CAPS+) can also undergo conversion (Fig 1b, S1b). Given that unmodified C constitutes the vast majority of the genome, even a very low conversion rate could result in a significant number of false-positive signals. Additionally, did you mean "conversation" or "conversion"?

Response: We appreciate the reviewer’s comment. We have revised the statement as follows: Direct conversion of 5mC and 5hmC to T will preserve the genomic complexity, thus leading to high sequencing quality. Additionally, we have corrected the typo, replacing "conversation" with "conversion."

3.  Is the global hmC quantification in neuronal and non-neuronal cells of the hippocampus by mass spectrometry consistent with the levels obtained from scCAPS+ sequencing (Fig 2a)?

Response: We appreciate the reviewer’s insightful comments. The scCAPS+ technique specifically quantifies 5hmC levels at CG sites, whereas mass spectrometry detects 5hmC across all CG, CA, CT, and CC sites. The 5hmCG modification levels we reported (22.04% in neurons vs. 9.29% in non-neuronal cells of the mouse hippocampus) align closely with previously published Joint-Seq data (25.6% in neurons vs. 9.5% in non-neuronal cells in the mouse cortex) (Fabyanic EB, et al., Nat Biotechnol. 2024. PMID: 37640946), supporting the accuracy of our 5hmC detection method. Furthermore, mass spectrometry studies have reported 5hmC levels of 0.65% in the cortex (Globisch D, et al., PLoS One. 2010. PMID: 21203455) and 0.53–0.77% in the hippocampus (Occean JR, et al., Nat Commun. 2024. PMID: 39069555), suggesting that neurons in both the cortex and hippocampus may exhibit similar levels of 5hmCG modification. Although no mass spectrometry data specific to neurons and non-neuronal cells in the hippocampus is available, the consistency between our 5hmCG data and Joint-Seq data, as well as the similarity in 5hmC levels between the cortex and hippocampus, indicate that our method reliably maps 5hmCG modifications in neuronal and non-neuronal cells within the mouse hippocampus.

4.  In Fig 1b and Fig S5b, the Y-axis labeling should be changed to "conversion rate." Additionally, the legend should specify the number of single cells used.

Response: We appreciate the reviewer’s comment. We have revised the Y-axis labels in Figures 1b and S5b (now Fig. S7b) to reflect "conversion rate" as suggested. Additionally, we have included the number of single cells utilized in the corresponding legend.

5. In Fig 2f, what does the scale bar represent?

Response: We appreciate the reviewer’s comment and apologize for any confusion. The scale bar represents the normalized gene body hmCG level, calculated using the NormalizeData function in the Seurat package with default parameters. We have now included this clarification in the corresponding figure legend.

6. In Fig S1b, how were the spike-ins adjusted for different cell input amounts?

Response: We appreciate the reviewer’s comment. For low input samples, we incorporate spike-ins as follows: 0.5% methylated lambda DNA, 0.1% 5-hydroxymethylcytosine (5hmC), and 0.1% unmethylated 2kb DNA for 5mC, 5hmC, and unmethylated controls, respectively. We estimate one cell to contain approximately 5 pg of genomic DNA. Thus, the spike-in amounts are adjusted based on cell input as follows: for 10 cells, we use 0.25 pg methylated lambda, 0.05 pg 5hmC, and 0.05 pg unmethylated 2kb; for 100 cells, 2.5 pg methylated lambda, 0.5 pg 5hmC, and 0.5 pg unmethylated 2kb; for 1,000 cells, 25 pg methylated lambda, 5 pg 5hmC, and 5 pg unmethylated 2kb; and for 10,000 cells, 250 pg methylated lambda, 50 pg 5hmC, and 50 pg unmethylated 2kb. 


Reviewer #4: The manuscript presents direct sequencing methodologies, scTAPS and scCAPS+, designed for the quantitative detection of 5mC and 5hmC at single-base resolution in single cells. These methods achieve high mapping efficiencies and demonstrate accurate profiling of 5mC and 5hmC in CD8+ T cells and mES cells. A notable finding is the global increase in 5hmC within the hippocampus of aging mice, observed in both neurons and non-neurons. Overall, the manuscript makes a significant contribution to the field of single-cell epigenomics by introducing new tools for the direct detection of 5mC and 5hmC.

Response: We would like to express our gratitude for the reviewer’s positive comments on our scTAPS/scCAPS+ manuscript. Please find the point-to-point response below.

A few comments:
1. The authors need to cite previously published methods, such as 6-letter sequencing (PMID: 36747096) and SIMPLE-seq (PMID: 38336903), which allow for the simultaneous analysis of 5mC and 5hmC at both bulk and single-cell levels, in the Introduction as well as the Discussion of this article.

Response: we appreciate the reviewer's suggestions. We have cited both methods in the introduction and discussion as requested. 
Line 54-56:
Notably, several newly developed methods, such as six-letter sequencing [13] and SIMPLE-seq [14], have recently emerged, allowing the simultaneous detection of both 5mC and 5hmC.
 Line 143-146:
The strengths and limitations of our methods become evident when compared to various current methods [7, 10, 13, 14] capable of detecting both 5mC and 5hmC modifications (Additional file 3).

2. The text and legend associated with Figure 1e mention the levels of 5mCG and 5hmCG along a 7 MB region of chr 2 in human CD8+ T cells (top) and a 117 MB region of Chr 12 in mouse ES cells but only provide data for one of the two modifications in each case. The authors should provide both 5mCG and 5hmCG data for both T cells and mouse ES cells. Also, it would be worthwhile to show a similar-sized (10-30 MB) region for the mouse ES cells, for instance the region currently at the very right of the mESC panel, since there is a greater range of 5hmC levels in this region.

Response: We appreciate the reviewer’s valuable suggestions. To address the reviewer’s concerns, we have replotted both the 5mCG (scTAPS) and 5hmCG (scCAPS+) data for the same region in mouse ES cells (Data #4). The results demonstrate a strong consistency between the merged single-cell and the bulk TAPS/CAPS+ data in mouse ES cells. Additionally, we have updated the IGV plot for scCAPS+ in mouse ES cells within a similarly sized region (chr12:81,000,000-111,000,000) (new Fig.1e, bottom), highlighting the clear consistency between the merged scCAPS+ data and the bulk CAPS+ data.

Data #4 to reviewer only
A close-up of a line

AI-generated content may be incorrect.
New Fig.1e, bottom
A close-up of a screen

AI-generated content may be incorrect.
3. In Figures 1d-f, the authors compare bulk and single-cell data, the results show little difference between the two. Can the authors strengthen the analysis by highlighting any unique insights provided by single-cell sequencing that are not apparent from bulk sequencing?

Response: We appreciate the reviewer’s comments. In Figure 1, our aim is to validate the scTAPS methodology in CD8+ T cells and the scCAPS+ methodology in mESCs. To address the reviewer’s concern, we conducted additional analysis of our scTAPS data to further demonstrate its ability to explore cellular heterogeneity in 5mC signals within CD8+ T cells. Specifically, we identified genes exhibiting either consistent or highly variable 5mC modifications patterns that could only be detected within single-cell. For instance, genes such as EXOC4 (exocyst complex component 4) have been implicated in vesicle transport processes (Li, et al., Mol Cancer Res, 2022, PMID: 35471457), while ARHGAP15, a Rac1-specific  GTPase Activating Protein, has been linked to T cell functional subpopulations at the isoform level (Liu S, et al., Sci Rep. 2021. PMID: 33674641). Both genes exhibit consistently high levels of 5mC modifications across different cells (Data #5).  Notably, several genes associated with immune cell activation and differentiation, including MXD4 (Vasilevsky NA, et al., Eur J Immunol. 2011, PMID: 21400495), CD47 (Nath PR, et al.,  Oncoimmunology. 2022, PMID: 36105746), VEGFA (Voron T, et al., J Exp Med. 2015, PMID: 25601652), ALKBH5 (Ge J, et al., Transl Oncol. 2023, PMID: 37224767), and TMED7 (Connolly DJ, et al., J Biol Chem. 2013, PMID: 23319592), display varied 5mCG modifications between cells (Data #5). These dynamic patterns, whether stable or variable, are insights uniquely captured by single-cell sequencing, which bulk sequencing does not reveal.

Data #5 to reviewer only
A screenshot of a graph

AI-generated content may be incorrect.
4. In Figure 2a, the authors observed a global increase in 5hmC within the hippocampus of aging mice, evident in both neurons and non-neurons. What is the expression status of TET1, TET2, and TET3 in these cells?

Response: we appreciate the reviewer’s comments. To address the reviewer’s concerns, we examined the expression levels of Tet1, Tet2, and Tet3 in the hippocampus of mice (3 months vs. 18 months) using data from the published Spatiotemporal Brain Map database (https://twc-stanford.shinyapps.io/spatiotemporal_brain_map/) (Data #6). The analysis shows no significant changes in the expression of these genes during aging. Additionally, a study (Chen H, et al., Restor Neurol Neurosci. 2012. PMID: 22426040) also reported an increase in 5hmC levels detected via ELISA in the aging hippocampus, without corresponding changes in Tet1, Tet2, or Tet3 expression. Further research suggests that a hyper-quiescent state in aging may contribute to 5hmC accumulation, even with stable expression and activity of TET1/2/3 (Occean JR, et al., Nat Commun. 2024. PMID: 39069555), which could explain the observed elevation of 5hmC modifications in aged hippocampal cells. 

Data #6 to reviewer only
A screenshot of a graph

AI-generated content may be incorrect.
5. The authors only have single-cell sequencing data for Figure 2, unlike Figure 1 which includes bulk sequencing data. It would be useful if the authors could supplement Figure 2 with bulk sequencing data to compare bulk and single-cell data and determine if there are specific findings in the single-cell data.

Response: We appreciate the reviewer’s comments. In the method validation section (Figure 1), we compared the results from single-cell and bulk sequencing, demonstrating the accuracy and consistency of our single-cell approach. Following this, we applied our scCAPS+ methodology to mice hippocampus cells. While we acknowledge that comparing bulk and single-cell data yields limited specific findings, we have tried to address the reviewer's concerns by further clustering the neurons based on our single-cell 5hmC data. Our analysis revealed two primary neuronal cell types: an excitatory neuron subtype, characterized by high hmCG levels on Sv2b, and an inhibitory neuron subtype, marked by high hmCG levels on Slc6a1 (Data #3). These genes, Sv2b (Pazarlar BA, et al., Mol Brain. 2022. PMID: 35578248) and Slc6a1 (Mermer F, et al., Brain. 2021. PMID: 34028503), are established markers for excitatory and inhibitory neurons, respectively, based on RNA expression profiles. This indicates that our methodology has potential for subtype classification, which bulk sequencing alone cannot provide.

Data #3 to reviewer only
A screenshot of a graph

AI-generated content may be incorrect.
6. Although the manuscript briefly compares scTAPS/scCAPS+ with SIMPLE-seq methods, a more detailed comparison of their bulk RNA-seq data across a wider range of methodologies including 6-letter sequencing and SIMPLE-seq techniques would provide a clearer picture of the advantages and potential limitations of the new methods.

Response: We appreciate the reviewer’s comments. To address the reviewer’s concerns, we compared current sequencing methods for both 5mC and 5hmC detection. The summarized table (new Additional file 3) shows the advantages and disadvantages regarding each method (six-letter-seq/Cabernet-seq/SIMPLE-seq/Joint-seq/scTAPS and scCAPS+). 

new Additional file 3

six-letter-seq
Füllgrabe J, 2023 
Cabernet -seq
Yunlong Cao, 2023
SIMPLE-seq 
Bai D, 2024
Joint-seq 
Emily B, 2024
scTAPS/CAPS+
(this study)
Target
5mC/5hmC 
5mC/5hmC 
5mC/5hmC 
5mC/5hmC 
5mC/5hmC 
Accuracy/
conversion 
95.24%/97.93 %
98.50%/99.70% 
87%/85.60% 
98%/98.80% 
96.60%/93.00% 
Bisulfite Treatment 
Bisulfite-free 
Bisulfite-free 
Bisulfite-free 
Bisulfite 
Bisulfite-free 
Detection Mechanism 
Indirect 
Indirect 
Direct 
Indirect 
Direct 
Simultaneous Detection 
Yes 
No 
Yes 
Yes (split) 
No 
 Resolution 
Bulk 
Single-cell 
Single-cell 
Single-cell 
Single-cell 
Throughput 
N/A 
High 
High 
Low 
Low
We also compared different single cell methods and have plotted the mapping rate (new supplemental figure, Fig. S3a), the correlation between the number of raw sequencing reads per cell and the CpG/genome coverage per cell (new supplemental figure, Fig. S3b). The results clearly demonstrate that scTAPS and scCAPS+ achieve higher mapping rates compared to indirect methods, and higher CpG and genomic coverage compared to snmC-seq2 (Luo C, et al., Nat Commun. 2018. PMID: 30237449), scBS-seq (Smallwood SA, et al., Nat Methods. 2014. PMID: 25042786), sci-Cabernet (Cao Y, et al., PNAS. 2023. PMID: 38011566), and Joint-snhmC-seq (Fabyanic EB, et al., Nat Biotechnol. 2024. PMID: 37640946) when assessed under comparable sequencing depths.

New Fig. S3a
A graph of a number of different types of numbers

AI-generated content may be incorrect.
New Fig. S3b
A graph of a function

AI-generated content may be incorrect.
Minor
1. The author profiles 5mC and 5hmC in CD8+ T cells and mouse ES cells in Figure 1. However, Figures 1b-f do not indicate whether the analyzed data is from T cells, mouse ES cells, or both cell types combined. Can the authors provide specific clarification?

Response: We appreciate the reviewer’s suggestions. We have now included specific indications of the cell types in the figure and related legends. This should provide clearer context for the reader and improve the overall clarity of the data presented. Thank you for your suggestion.


2. Line 124: It should be 'SIMPLE-seq,' not 'SAMPLE -seq.

Response: Thank you for pointing out the typo. It has been corrected to "SIMPLE-seq" in new line 131 and 133.

2nd round
Reviewer 1
I read the revised manuscript. I have no additional comments. The authors have adequately addressed my questions.

Reviewer 4
The authors have satisfactorily addressed al our concerns
